# Diagnostic Delays in Thoracic Cancer Care: A Data-Linkage, Cohort Study between Primary Care, Hospital, and Registry Data

**DOI:** 10.34133/hds.0457

**Published:** 2026-05-04

**Authors:** Jianrong Zhang, Maarten J. IJzerman, Damien McCarthy, Rebecca J. Bergin, Chris Kearney, Sally Philip, Alex Lee, Xiaofei Wang, Benjamin Solomon, Marliese Alexander, Gavin M. Wright, Jon D. Emery

**Affiliations:** ^1^ Department of General Practice and Primary Care & Collaborative Centre for Genomic Cancer Medicine, Faculty of Medicine, Dentistry and Health Sciences, University of Melbourne, Melbourne, Victoria, Australia.; ^2^ Victorian Comprehensive Cancer Centre, Melbourne, Victoria, Australia.; ^3^ Analysis of Linked Information on Cancer, Victorian Cancer Registry, Cancer Council Victoria, Melbourne, Victoria, Australia.; ^4^ Cancer Health Services Research Unit, Centre for Health Policy, School of Population and Global Health, Faculty of Medicine, Dentistry and Health Sciences, University of Melbourne, Melbourne, Victoria, Australia.; ^5^ Erasmus School of Health Policy and Management, Rotterdam, The Netherlands.; ^6^ Centre for Quality and Patient Safety Research, Institute for Health Transformation, School of Nursing and Midwifery, Faculty of Health, Deakin University, Geelong, Victoria, Australia.; ^7^ Cancer Epidemiology Division, Cancer Council Victoria, Melbourne, Victoria, Australia.; ^8^ Department of Biostatistics and Bioinformatics, Duke University School of Medicine, Durham, NC, USA.; ^9^ Department of Medical Oncology, Peter MacCallum Cancer Centre, Melbourne, Victoria, Australia.; ^10^ Sir Peter MacCallum Department of Oncology, University of Melbourne, Melbourne, Victoria, Australia.; ^11^ Pharmacy Department, Peter MacCallum Cancer Centre, Melbourne, Victoria, Australia.; ^12^ Department of Cardiothoracic Surgery, St Vincent’s Hospital Melbourne, Melbourne, Victoria, Australia.; ^13^ Department of Surgery, University of Melbourne, Melbourne, Victoria, Australia.; ^14^ Division of Cancer Surgery, Peter MacCallum Cancer Centre, Melbourne, Victoria, Australia.; ^15^ Population and Global Health, Lee Kong Chian School of Medicine, Nanyang Technological University, Singapore, Singapore.

## Abstract

**Background:** Nearly half of patients with thoracic cancer are diagnosed at a late stage, contributing to poor survival. This may be due to delays in diagnosis. **Methods:** This study established a longitudinal cohort by linking data between primary care, hospital, and registry datasets in the State of Victoria, Australia, and evaluated the diagnostic intervals (DIs) against the recommended time frames in the Australian Optimal Care Pathways (OCP) guidelines. The intervals included the DI, the time from the first presentation in primary care to cancer diagnosis at hospitals (OCP recommendation, 35 d); and the diagnostic and treatment interval (DTI), from the first presentation in primary care to cancer treatment initiation (OCP recommendation, 49 d). **Results:** A total of 268 patients diagnosed between 2005 and 2021 were linked and analyzed for DI, and 249 for DTI. Among them, 76% experienced diagnostic delay, with DI longer than the recommended 35 d; 86% experienced diagnostic and treatment delay, with DTI longer than the recommended 49 d. Multivariable logistic regression analyses found both diagnostic delays to be consistent across the observation period (*P* > 0.05). Diagnostic delay was associated with any comorbidity (odds ratio [OR] = 2.49 [95% confidence interval, 1.23 to 5.05]), existing respiratory disease (OR = 2.51 [1.30 to 4.83]), and stages I and II (OR = 3.89 [1.55 to 9.77]). **Conclusion:** This study demonstrates the feasibility of conducting research with linkage to primary care datasets in Australia. We demonstrated healthcare system delays, which may contribute to poorer thoracic cancer outcomes.

## Introduction

The healthcare system in Australia is well recognized as one of the best in the world, particularly in terms of healthcare access and quality [1], as well as effective coverage of health services [2]. The universal healthcare system comprises high-quality and cost-effective primary and specialist care, supported by a taxpayer-funded public health insurance scheme for fee-free treatment in public hospitals [3]. In the Australian system, typically, most patients attend a primary care physician (PCP) first, who serves as a gatekeeper to disease specialist care. Australian PCPs have direct access to a range of investigations, including blood tests and radiology such as chest x-ray and computed tomography. Patients with suspected cancer can be referred to fully funded public hospital specialists or private specialists with partial coverage of this through Medicare, Australia’s universal health insurance scheme.

For thoracic cancer, even though it is the leading cause of cancer death globally and in Australia [4,5], Australia has relatively better mortality and survival rates compared to the global estimates [6,7]. Even so, similar to many other countries, over half of the patients in Australia are still diagnosed at a late stage [8,9], losing opportunities for curable treatment [10,11]. Australia and the rest of the world have still shared a crucial challenge for decades: diagnostic and treatment delays in thoracic cancers. The World Health Organization strongly advocates reducing cancer diagnosis delays [12]. In Australia, diagnostic delays are recognized as a primary care cancer research priority [13]. To guide potential interventions addressing the delays, it is essential to investigate the timeliness of thoracic cancer diagnosis and treatment comprehensively. The COVID-19 pandemic in recent years has made such an investigation more critical than ever [14].

The Australian guidelines “*Optimal care pathway for people with lung cancer*” (Optimal Care Pathways) was first published in 2014 and now is in the second edition published in 2021, with the recommended maximum time frames of the cancer diagnosis and treatment to guide the current clinical practice [15]. According to the guidelines, patients should receive test results no more than 1 week after presenting to primary care, have a specialist appointment no more than 2 weeks after the primary care referral, and have completed diagnostic tests no more than 2 weeks after the specialist appointment [15]. In other words, patients in Australia should experience less than 35 d from their first presentation in primary care to the date of cancer diagnosis at the hospitals. The guidelines also recommend that patients should receive cancer treatment no more than 6 weeks after the specialist referral [15], adding 14 d between cancer diagnosis and treatment within the care pathway. In total, patients should experience less than 49 d from the first presentation to the date of cancer treatment initiation. However, the country lacks real-world evidence from large-scale datasets for these time intervals from the entry point in primary care—the setting where the vast majority of patients first present within the Australian healthcare system.

Therefore, we conducted a first-ever data linkage between primary care, hospital, and registry datasets in Australia, aiming to evaluate thoracic cancer timeliness starting from primary care. In this article, we describe the study design and study cohort characteristics, demonstrate the extent of delays patients experience for thoracic cancer diagnosis and treatment against the recommended time frames outlined in the Australian guidelines, and investigate any factors explaining the diagnostic delays based on patients’ demographic and clinical characteristics.

## Methods

This is a cohort study, with data linkage between primary care, hospital, and registry datasets to establish the study cohort and key variables for analysis. Study design and reporting were informed by the Aarhus statement [16], our newly proposed framework on time intervals [17], the REporting of studies Conducted using Observational Routinely collected health Data (RECORD) statement [18], and the REST guideline [19].

### Data source, linkage, and patient selection

This study was possible using the Victorian Comprehensive Cancer Centre (VCCC) Data Connect platform. The Data Connect platform provides a large-scale, comprehensive data linkage between detailed primary care, hospital, and cancer registry data for patients with cancer for the first time in Australia [20]. The data access, linkage, and related ethical approval process are based on collaborations between the VCCC Alliance (including the Peter MacCallum Cancer Centre and St Vincent’s Hospital), BioGrid Australia, and the University of Melbourne, achieved using a deidentification and privacy-protected linkage service by BioGrid’s data access and sharing infrastructure, and researched via the Secure Research Environment housed at the University of Melbourne.

A more detailed description of the platform, including its capacity and data linkage with multiple datasets, can be found in our publication and other resources elsewhere [20–24]. Regarding the data linkage for this study, in brief, it was achieved via the Unique Subject Identifier (USI), a unique key encrypted for each patient. For primary care datasets, a USI was generated by the software GRHANITE. GRHANITE incorporated a cryptographic mechanism to extract data from any person-identifiable dataset and then created an encrypted key for each patient that allowed data linkage with other datasets without involving patients’ identifiers, including the names of patients or practitioners [22–24]. For hospital datasets, USI was generated relying on a cryptographic mechanism by BioGrid [20]. Through this data linkage, studies can investigate the patient journey at any milestone within the cancer continuum from prediagnosis to posttreatment periods.

**Fig. 1.**
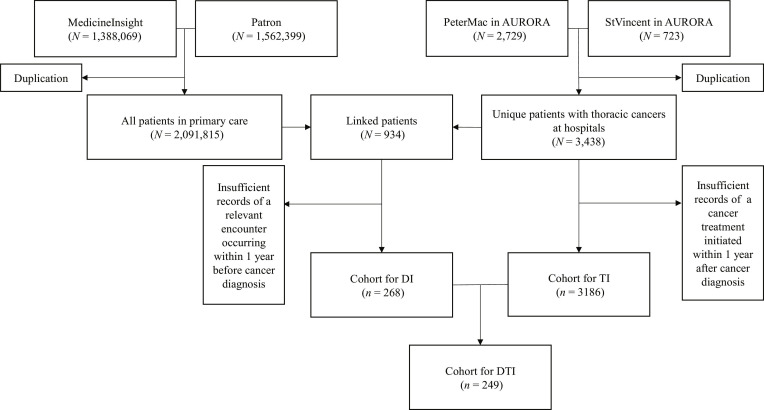
Data linkage between primary care and registry dataset. PeterMac, Peter MacCallum Cancer Centre; StVincent, St Vincent’s Hospital; AURORA, AUstralian Registry and biObank of thoRAcic cancers; DI, diagnostic interval; DTI, diagnostic and treatment interval; TI, treatment interval.

The establishment of this study cohort and the time intervals was based on 2 primary care datasets, MedicineInsight [25] and Patron [26], and thoracic-cancer-specific datasets from 2 hospitals, Peter MacCallum Cancer Centre and St Vincent’s Hospital, via the Australian Registry and biObank of thoRAcic cancers (AURORA) (Fig. 1) [27]. MedicineInsight is the first nationwide primary care data program now managed by the Australian Commission on Safety and Quality in Health Care via data extraction from the clinical software used in general practices [25]. Its Victorian subset was used for this study, involving 1.39 million patients from 92 general practices in 2007–2017 [20]. Patron is the data repository for the Data for Decisions program managed by the Department of General Practice and Primary Care at the University of Melbourne. The data were collected from deidentified health electronic records of general practices in Victoria with the permission of the primary care providers [26]. In Patron, 1.56 million patients from 130 Victorian general practices in 1996–2020 were accessible for this study [20]. AURORA is a multisite clinical registry that captures real-world data on patients with thoracic cancers in Australia [27]. Specific to this study, the AURORA provided access to 2,729 patients diagnosed in 1984–2021 at Peter MacCallum Cancer Centre and 723 patients at St Vincent’s Hospital in the State of Victoria.

This project received ethical approval from the Melbourne Health Human Research Ethics Committee in 2020 (BioGrid project number: 202005/8). The VCCC Data Connect Program involves patients and the public through representation on its Steering Committee and regular meetings to discuss specific projects, including those investigating diagnostic delays.

The inclusion criteria for the data-linkage cohort to investigate diagnostic intervals (DIs) were as follows: (a) Patients were diagnosed with any thoracic cancer (e.g., non-small-cell lung cancer [NSCLC], small-cell lung cancer, other neuroendocrine tumors, and mesothelioma) as recorded in the hospital datasets via the thoracic-cancer-specific registry (AURORA); (b) patients were recorded in both the primary care and hospital datasets; (c) patients had at least one recorded primary care encounter in the primary care datasets; (d) the primary care encounter(s) should be valid, defined as having a recorded date, occurring within 1 year before the date of thoracic cancer diagnosis at the hospitals, and being relevant to the diagnosis according to the Australian guidelines “*Evidence report for investigating symptoms of lung cancer: A guide for all health professionals*” [28]; (e) the date of clinical and/or pathological diagnosis at the hospitals for thoracic cancer should be recorded, and the cancer treatment type and the date of the treatment initiation should be recorded in the hospital datasets.

We included any thoracic cancer rather than limiting the study to specific histopathological types. We chose to do this because of the commonality of symptoms and signs at presentation.

### Outcome and exposure

The details of all variables, including their categories and sources, are shown in Table S1. The outcomes were the DI, from the date of the first presentation in primary care to the date of cancer diagnosis; and the diagnostic and treatment interval (DTI), from the date of the first presentation in primary care to the date of cancer treatment initiation [16,17]. The measures were binary variables: diagnostic delay, based on the length of DI against the recommended 35 d (longer or not); and diagnostic and treatment delay, based on the length of DTI against the recommended 49 d (longer or not), according to the Optimal Care Pathways guidelines [15]. The definitions of DI and DTI were in concordance with a revised framework [17] of time intervals within the cancer care pathways, which aims to address the lack of generalizability in using time intervals across countries [29], as developed relying on 2 previous frameworks supported in the Aarhus statement on reporting studies of time to cancer diagnosis [16]. The selection of these time intervals was based on how they commonly represent the patient journey within the healthcare system from the first presentation to cancer treatment initiation, as well as their widespread use in previous studies [30].

Many efforts were made to identify the first relevant encounter that occurred in primary care within 1 year before the date of thoracic cancer diagnosis at the hospitals. The relevance of the encounters was identified by screening the extensive records of primary care encounter information and matching specific symptoms and signs with the Australian guidelines “*Evidence report for investigating symptoms of lung cancer: A guide for all health professionals*” (see Table S2 for a full list of symptoms and signs) [28]. The encounter information was categorized as (a) reason for visit (converted to relevant symptom or sign as output), (b) request for chest radiology by PCPs, (c) observations on body weight (converted to weight loss as any subsequent observed weight more minor than the first observed weight), and (d) blood test results on platelet/thrombocyte count (converted to thrombocytosis if the platelet/thrombocyte count was over 400). To identify the relevant encounters, the decision to use a 1-year period preceding the disease diagnosis was based on previously reported diagnostic window studies of thoracic cancer [31–33].

This study also aims to understand any disease- and patient-level factors associated with the diagnostic delays. For the investigation, exposure variables included demographic characteristics (sex, age at diagnosis, year of thoracic cancer diagnosis, and ethnicity), environmental and behavioral characteristics (tobacco use, marijuana use, asbestos exposure, and alcoholism), comorbidities (any or never, cardiovascular diseases, respiratory diseases, neoplastic comorbidities, renal insufficiency, and diabetes), and clinical characteristics (histopathological type, stage at diagnosis, first encounter type of symptom or sign, radiology request, observed weight loss, and thrombocytosis).

### Statistical analysis

We defined the proportion of patients experiencing diagnostic delays when their DI was greater than 35 d and diagnostic and treatment delays when their DTI was greater than 49 d. Second, we conducted a multivariable logistic regression model analysis to evaluate the associations between patient characteristics and delays. The analysis was conducted with no adjustment (model 1); adjustment for sex and age (model 2); sex, age, and histopathology (model 3); sex, age, histopathology, and year of diagnosis (model 4); and sex, age, histopathology, year of diagnosis, ethnicity, and hospital site (model 5). The design of the multiple models was prespecified, and the rationale for choosing these variables in the statistical adjustment was based on their impact as potential confounders or covariates on the associations between the exposures and the outcomes. While the family-wise type I error rate will not be controlled through multiple testing methods, the results of statistical significance will be interpreted and carefully discussed on the basis of the consistencies of both the direction and significance of effect sizes across all the regression models being taken into account.

The above analyses were done for the whole cohort with any thoracic cancer and the subcohort with NSCLC—the most common thoracic cancer—to examine any inconsistency between the results. For the whole cohort, another subgroup analysis was conducted among patients with their first encounters of symptoms/signs and radiology request types, respectively; an extra analysis was conducted among patients diagnosed in 2011–2019 to mitigate the potential biased effect of the small number of patients diagnosed before 2011 and after 2019.

Last, to understand the generalizability of the analyzed results based on the included patients, we checked if there could be any differences in the characteristics of treated patients who were linked and eligible for analysis versus those who were not linked but were also treated in the same facilities. For this, a chi-squared test was conducted on the number of patients; patients in this analysis should have been diagnosed within the same period (2005–2021) and have valid records of cancer treatment type as well as the dates of diagnosis and treatment initiation (Table S3). In all the above analyses, statistical significance was indicated when the *P* value was lower than 0.05.

## Results

Through data linkage based on a total of 1,388,069 and 1,562,399 patients with all diseases in 2 primary care datasets as well as 2,729 and 723 patients with thoracic cancers in 2 hospital datasets, we included 268 and 249 patients diagnosed with any thoracic cancer in 2005–2021, respectively, for DI and DTI (Fig. 1). Among both the DI and DTI cohorts, 43% had stage IV (close to the national level—42%—including unknown stage) [9]; 82% had NSCLC (Table S4).

Table S5 details the first encounter type. Symptoms or signs were the main encounter type, accounting for 44% and 45% of the DI and DTI cohorts, respectively, followed by the radiology request (40% for DI and 39% for DTI). Among patients with the first encounter as a symptom or sign, 26% and 28% had infection or pneumonia, respectively, for DI and DTI. Among patients with the first encounter as a radiology request (106 for DI and 96 for DTI), the majority were x-ray request (66% for DI and 68% for DTI).

**Fig. 2.**
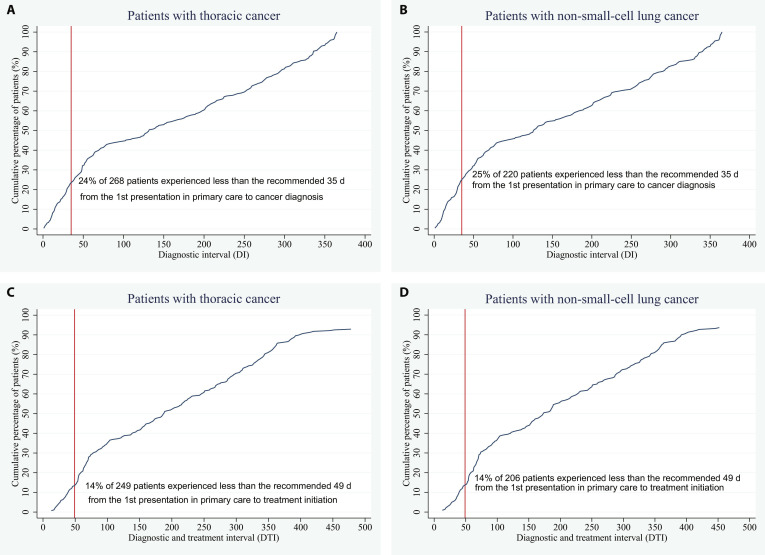
Pareto plot for diagnostic intervals (DIs). (A) DI in patients with any thoracic cancer; (B) DI in patients with non-small-cell lung cancer (NSCLC); (C) diagnostic and treatment interval (DTI) in patients with any thoracic cancer; (D) DTI in patients with NSCLC.

Table 1 presents the proportions of patients with diagnostic delays for any thoracic cancer, and Table S6 presents them for NSCLC. Overall, 76% of the 268 patients experienced diagnostic delay, with DI greater than the recommended 35 d; 86% of the 249 patients experienced diagnostic and treatment delay, with DTI longer than the recommended 49 d (Fig. 2). Among these, the proportions were slightly larger in patients aged over 75 years, histopathological types other than NSCLC, stages I and II, the first encounter of thrombocytosis, and the first treatment of both systemic therapy and radiotherapy used on the same day (Table 1).

**Table 1.** Patient characteristics in data-linkage cohorts with any thoracic cancer

Characteristics	Cohort for diagnostic interval (DI)			Cohort for diagnostic and treatment interval (DTI)		
	Delays (>35 d) (*N* = 205; 76%)	No delays (≤35 d) (*N* = 63; 24%)	Total (*N* = 268)	Delays (>49 d) (*N* = 213; 86%)	No delays (≤49 d) (*N* = 36; 14%)	Total (*N* = 249)
Demographic characteristics						
Sex						
Female	84 (76%)	27 (24%)	111	88 (85%)	15 (15%)	103
Male	121 (77%)	36 (23%)	157	125 (86%)	21 (14%)	146
Age at diagnosis						
<65	67 (69%)	30 (31%)	97	75 (79%)	20 (21%)	95
≥65 and <75	73 (78%)	20 (22%)	93	75 (86%)	12 (14%)	87
≥75	65 (83%)	13 (17%)	78	63 (94%)	4 (6%)	67
Year of diagnosis						
2005–2011	18 (72%)	7 (28%)	25	20 (91%)	2 (9%)	22
2012–2015	91 (78%)	25 (22%)	116	92 (85%)	16 (15%)	108
2016–2019	84 (76%)	27 (24%)	111	89 (86%)	15 (14%)	104
2020–2021	12 (75%)	4 (25%)	16	12 (80%)	3 (20%)	15
Ethnicity						
White	186 (77%)	57 (23%)	243	194 (86%)	32 (14%)	226
Other	19 (76%)	6 (24%)	25	19 (83%)	4 (17%)	23
Environmental and behavioral exposure						
Tobacco use						
Never	26 (74%)	9 (26%)	35	27 (82%)	6 (18%)	33
Past	116 (73%)	42 (27%)	158	123 (85%)	22 (15%)	145
Current	49 (80%)	12 (20%)	61	50 (88%)	7 (12%)	57
Missing	NA	NA	14	NA	NA	14
Marijuana						
Never	153 (76%)	49 (24%)	202	160 (86%)	26 (14%)	186
Current and past	20 (65%)	11 (35%)	31	21 (72%)	8 (28%)	29
Missing	NA	NA	35	NA	NA	34
Asbestos						
No	134 (77%)	41 (23%)	175	141 (87%)	22 (13%)	163
Yes	28 (61%)	18 (39%)	46	16 (89%)	2 (11%)	44
Unknown	NA	NA	22	NA	NA	18
Missing	NA	NA	25	NA	NA	24
Alcoholism						
No	165 (73%)	60 (27%)	225	177 (84%)	33 (16%)	210
Yes	16 (84%)	3 (16%)	19	14 (88%)	2 (12%)	16
Unknown	NA	NA	24	NA	NA	23
Comorbidities						
Overall						
None	31 (57%)	23 (43%)	54	39 (74%)	14 (26%)	53
At least one comorbidity	157 (80%)	40 (20%)	197	158 (88%)	21 (12%)	179
Other ^a^	NA	NA	17	NA	NA	17
Cardiovascular diseases						
No	108 (72%)	41 (28%)	149	116 (82%)	25 (18%)	141
Yes	97 (82%)	22 (18%)	119	97 (90%)	11 (10%)	108
Respiratory diseases						
No	94 (67%)	47 (33%)	141	108 (80%)	27 (20%)	135
Yes	93 (85%)	16 (15%)	109	88 (92%)	8 (8%)	96
Unknown	NA	NA	18	NA	NA	18
Neoplastic comorbidities						
No	158 (76%)	50 (24%)	208	168 (84%)	31 (16%)	199
Yes	47 (78%)	13 (22%)	60	45 (90%)	5 (10%)	50
Renal insufficiency						
No	193 (76%)	60 (24%)	253	201 (86%)	33 (14%)	234
Yes	12 (80%)	3 (20%)	15	12 (80%)	3 (20%)	15
Diabetes						
No	138 (72%)	55 (28%)	193	148 (84%)	29 (16%)	177
Yes	43 (84%)	8 (16%)	51	43 (88%)	6 (12%)	49
Unknown	NA	NA	24	NA	NA	23
Clinical status						
Histopathological type						
NSCLC	165 (75%)	55 (25%)	220	177 (86%)	29 (14%)	206
Other	37 (82%)	8 (18%)	45	34 (83%)	7 (17%)	41
Missing	NA	NA	3	NA	NA	2
Stage						
I–II	70 (92%)	6 (8%)	76	66 (93%)	5 (7%)	71
III–IV	127 (69%)	57 (31%)	184	142 (82%)	31 (18%)	173
Missing	NA	NA	8	NA	NA	5
1st primary care encounter type						
Symptom or sign	90 (77%)	27 (23%)	117	95 (86%)	16 (14%)	111
Radiology request	72 (68%)	34 (32%)	106	77 (80%)	19 (20%)	96
Observed weight loss	35 (95%)	2 (5%)	37	33 (97%)	1 (3%)	34
Thrombocytosis	8 (100%)	0	8	8 (100%)	0	8
1st cancer treatment						
Systemic therapy	NA	NA	NA	49 (82%)	11 (18%)	60
Surgery	NA	NA	NA	52 (90%)	6 (10%)	58
Radiotherapy	NA	NA	NA	77 (82%)	17 (18%)	94
Systemic therapy + radiotherapy ^b^	NA	NA	NA	35 (95%)	2 (5%)	37

NSCLC, non-small-cell lung cancer; NA, not applicable.

^a^ The rest after excluding those with at least one comorbidity and those without any comorbidity.

^b^ Both systemic therapy and radiotherapy were initiated on the same day.

Table 2 presents the results of the associations between patient characteristics and delays, adjusted for sex, age, histopathological type, year of diagnosis, ethnicity, and hospital site. For the diagnostic delay based on DI, risk factors for the delay consistently identified in all 5 models were any comorbidity (odds ratio [OR] = 2.49 [95% confidence interval, 1.23 to 5.05]), preexisting respiratory disease (OR = 2.51 [1.30 to 4.83]), and stages I and II (OR = 3.89 [1.55 to 9.77]). Asbestos exposure was associated with less likelihood of diagnostic delay (OR = 0.43 [0.20 to 0.89]) (Table 2 and Table S7). In patients with NSCLC, additional factors for longer DI included the comorbidity of diabetes (OR = 3.03 [1.10 to 8.30]); less likelihood of diagnostic delay was associated with radiology request (OR = 0.41 [0.21 to 0.82]) compared to symptom or sign as the first encounter type (Table 2 and Table S8). However, the above factors were not associated with the diagnostic and treatment delay (in terms of DTI), except for any comorbidity (OR = 3.61 [1.42 to 9.16]) among patients with NSCLC (Table 2 and Tables S9 and S10). Similar findings were observed among patients diagnosed in the pre-COVID period 2011–2019 (Tables S11 and S12). None of the results suggested significant associations between the year of diagnosis and risk of the diagnostic delays (Table 2 and Tables S7 to S12).

**Table 2.** Associations between characteristics and diagnostic delays

Characteristics	DI > 35 d		DTI > 49 d	
	Thoracic cancer (OR)	NSCLC (OR)	Thoracic cancer (OR)	NSCLC (OR)
Demographic characteristics				
Sex: male versus female	0.97 (0.53–1.75)	1.04 (0.55–1.97)	0.91 (0.43–1.91)	0.97 (0.43–2.21)
Age at diagnosis				
<65	0.58 (0.29–1.13)	0.57 (0.27–1.20)	0.58 (0.26–1.28)	0.42 (0.17–1.07)
≥65 and <75	1.00	1.00	1.00	1.00
≥75	1.37 (0.62–3.05)	1.31 (0.57–2.99)	2.44 (0.73–8.08)	1.97 (0.55–6.99)
Year of diagnosis				
2005–2011	1.02 (0.37–2.78)	0.96 (0.32–2.87)	2.34 (0.47–11.54)	1.61 (0.32–8.23)
2012–2015	1.32 (0.69–2.52)	1.31 (0.65–2.63)	1.05 (0.48–2.31)	1.03 (0.42–2.154)
2016–2019	1.00	1.00	1.00	1.00
2020–2021	0.96 (0.27–3.33)	0.57 (0.14–2.24)	0.67 (0.16–2.74)	0.29 (0.06–1.40)
Ethnicity: White/Caucasian versus other	1.12 (0.41–3.09)	1.60 (0.48–5.34)	1.04 (0.32–3.39)	2.09 (0.42–10.41)
Environmental and behavioral exposure				
Tobacco use				
Never	1.00	1.00	1.00	1.00
Past	0.92 (0.36–2.36)	1.19 (0.45–3.13)	1.07 (0.35–3.32)	1.45 (0.45–4.67)
Current	1.65 (0.56–4.83)	1.58 (0.52–4.82)	1.72 (0.47–6.26)	1.87 (0.48–7.27)
Marijuana: current or past versus never	0.62 (0.25–1.52)	0.49 (0.18–1.35)	0.57 (0.20–1.59)	0.37 (0.12–1.13)
Asbestos: yes versus no	0.43 (0.20–0.89)*	0.41 (0.18–0.93)*	0.44 (0.19–1.06)	0.50 (0.19–1.37)
Alcoholism: yes versus no	1.97 (0.52–7.50)	1.66 (0.32–8.64)	1.63 (0.33–8.15)	1.81 (0.20–16.21)
Comorbidities				
Any comorbidities: at least one versus none	2.49 (1.23–5.05)*	3.72 (1.70–8.12)*	2.03 (0.87–4.71)	3.61 (1.42–9.16)*
Cardiovascular diseases: yes versus no	1.81 (0.94–3.48)	2.51 (1.23–5.14)*	1.86 (0.80–4.34)	2.93 (1.10–7.80)*
Respiratory diseases: yes versus no	2.51 (1.30–4.83)*	2.47 (1.22–5.00)*	2.21 (0.93–5.24)	2.47 (0.93–6.56)
Neoplastic comorbidities: yes versus no	1.14 (0.55–2.35)	1.27 (0.58–2.80)	1.44 (0.51–4.06)	2.45 (0.68–8.83)
Renal insufficiency: yes versus no	1.20 (0.32–4.53)	1.80 (0.38–8.60)	0.50 (0.13–1.96)	0.71 (0.14–3.58)
Diabetes: yes versus no	1.93 (0.84–4.48)	3.03 (1.10–8.30)*	1.10 (0.41–2.97)	2.01 (0.55–7.26)
Clinical status				
Histopathological type: other versus NSCLC	1.84 (0.79–4.28)	NA	0.95 (0.37–2.38)	NA
Stage: I–II versus III–IV	3.89 (1.55–9.77)*	4.51 (1.66–12.25)*	2.14 (0.74–6.18)	2.97 (0.83–10.58)
1st primary care encounter type				
Symptom or sign	1.00	1.00	1.00	1.00
Radiology request (CT or x-ray)	0.49 (0.26–0.91)*	0.41 (0.21–0.82)*	0.58 (0.27–1.24)	0.55 (0.23–1.30)
Observed weight loss	4.41 (0.97–20.04)	3.54 (0.76–16.51)	4.66 (0.58–37.29)	3.80 (0.45–31.93)
Thrombocytosis	NA	NA	NA	NA
1st cancer treatment				
Systemic therapy	NA	NA	1.00	1.00
Surgery	NA	NA	1.11 (0.33–3.76)	1.20 (0.30–4.80)
Radiotherapy	NA	NA	0.74 (2.29–1.85)	0.64 (0.22–1.88)
Systemic therapy + radiotherapy ^a^	NA	NA	3.42 (0.66–17.61)	3.28 (0.61–17.76)

Data in parentheses are 95% confidence intervals. Adjustment: sex, age, histopathological type (for cohorts with any thoracic cancer), year of diagnosis, ethnicity, and hospital site.

OR, odds ratio; CT, computed tomography; NSCLC, non-small-cell lung cancer; NA, not applicable; DI, diagnostic interval; DTI, diagnostic and treatment interval.

* Statistically significant, as indicated by the *P* value lower than 0.05.

^a^ Both systemic therapy and radiotherapy were initiated on the same day.

A recording of pneumonia or respiratory tract infection as the first encounter was associated with the delays in terms of DI (OR = 6.05 [1.04 to 35.25]) and DTI (OR = 9.52 [1.19 to 76.13]) compared to patients with cough (Table 3 and Tables S13 and S14). Detailed results from all adjustment models are available in Tables S7 to S14.

**Table 3.** Subgroup analysis on associations between first primary care encounter types and diagnostic delays in patients with any thoracic cancer

1st primary care encounter	Cohort for DI				Cohort for DTI			
	Delays (>35 d)	No delays (≤35 d)	Total	OR (95% CI)	Delays (>49 d)	No delays (≤49 d)	Total	OR (95% CI)
Symptom or sign								
Pneumonia or infection	27 (87%)	4 (13%)	31	6.05 (1.04–35.25)*	29 (94%)	2 (6%)	31	9.52 (1.19–76.13)*
COPD/COAD	20 (100%)	0 (0%)	20	NA	17 (100%)	0 (0%)	17	NA
Pain	9 (69%)	4 (31%)	13	1.71 (0.25–11.52)	10 (83%)	2 (17%)	12	2.86 (0.31–26.89)
Cough	4 (44%)	5 (56%)	9	1	5 (56%)	4 (44%)	9	1
Bronchitis	6 (75%)	2 (25%)	8	1.72 (0.17–17.72)	7 (88%)	1 (12%)	8	3.10 (0.19–51.71)
Asthma	5 (71%)	2 (29%)	7	2.39 (0.26–21.70)	6 (86%)	1 (14%)	7	4.68 (0.34–64.54)
Other	19 (66%)	10 (34%)	29	1.26 (0.23–6.74)	21 (78%)	6 (22%)	27	1.74 (0.28–10.69)
Radiology								
X-ray	52 (74%)	18 (26%)	70	1	54 (83%)	11 (17%)	65	1
CT	18 (58%)	13 (42%)	31	0.51 (0.19–1.37)	21 (78%)	6 (22%)	27	0.69 (0.21–2.24)
Both on the same date	2 (40%)	3 (60%)	5	NA	2 (50%)	2 (50%)	4	NA

Adjustment: sex, age, histopathological type, year of diagnosis, ethnicity, and hospital site.

CI, confidence interval; COPD, chronic obstructive pulmonary disease; COAD, chronic obstructive airways disease; DI, diagnostic interval; DTI, diagnostic and treatment interval; NA, not applicable; CT, computed tomography.

* Statistically significant, as indicated by the *P* value lower than 0.05.

## Discussion

This study applied the first linkage between primary care, hospital, and clinical cancer registry data in Australia, providing evidence of the timeliness from the first presentation in primary care to thoracic cancer diagnosis and treatment. Key findings include 76% of the cohort having a diagnostic delay and 86% delayed start to treatment when benchmarked against the recommended time frames in the Australian Optimal Care Pathways [15]. We also studied various patient and clinical characteristics to determine their associations with the delays. In particular, any comorbidity, preexisting respiratory disease, or diabetes was associated with diagnostic delay. Other risk factors included patients with earlier stages (I and II) and those with pneumonia or respiratory tract infection as the first reason for encounter. In contrast, patients with asbestos exposure, radiology request as the first encounter, were less likely to experience a diagnostic delay.

Our results align with evidence supporting a long wait for thoracic cancer diagnosis and treatment, as summarized in an overview of systematic and scoping reviews we previously conducted [30]. This study also included an analysis of time periods and found that the magnitude of delays was not statistically different between consecutive periods in 2005–2021. Notably, the comparison did include the years (2020 and 2021) of the COVID-19 pandemic in which 75% of patients experienced diagnostic delay and 80% experienced diagnostic and treatment delay. No difference between the COVID-19 years and before was also found in a New South Wales study, but it reported only the interval from specialist referral to radiotherapy initiation [34]. Given our results, an important implication is that diagnostic delays have persisted over time without showing a sign of significant improvement. In addition, the Australian guidelines [15] may have underestimated the real-world conditions in terms of the time required to diagnose and treat thoracic cancers, starting from the first presentation in primary care.

It is vital to understand the factors contributing to delays in diagnosis and treatment so that potential interventions can be implemented to address the issue. Our current results on the associations of delays with certain patient characteristics explain the difficulties in symptom-based diagnosis for thoracic cancers, especially for patients with comorbidities or at earlier stages of the disease. Usually, early-stage thoracic cancers have no symptoms or lack typical alarm symptoms [31,35,36], making awareness difficult for patients to seek help [37–39] and for physicians who are less likely to initiate further investigations [39,40]. Regarding early-stage symptoms and signs, in fact, thoracic cancers share similarities with many diseases, such as cough, chest and shoulder pain, shortness of breath, chest infection, and fatigue. These are very common in other respiratory diseases (e.g., asthma, chronic obstructive pulmonary disease, and pneumonia) and cardiovascular diseases (e.g., coronary artery disease) [31,36]. More importantly, all of these symptoms have positive predictive values of only about 1% for diagnosing lung cancer [41]. As a result, minimization or misattribution of symptoms and signs is easily made by patients and physicians [37–40].

Asbestos exposure was associated with less diagnostic delay. In Australia, workers experiencing asbestos exposure are required to undergo health monitoring. Greater awareness of the risks of asbestos could prompt earlier presentation and referral for symptoms of thoracic cancer [42], facilitating earlier diagnosis and treatment.

We also found radiology request as the first encounter to be associated with a lower risk of diagnostic delay in patients with NSCLC. Before concluding on the value of early radiology use in addressing the delays, further research is needed to understand the reasons for the radiology request in this cohort. It is possible that other prior consultations with relevant symptoms or signs were not captured in the 2 primary care datasets or were made beyond the currently defined diagnostic window (within 1 year before cancer diagnosis at hospitals), potentially biasing the current results.

Australia has recently launched a national lung cancer screening program for high-risk populations, but symptomatic presentation is likely to remain the main route to diagnosis and treatment [43]. Additional efforts will be necessary to improve the timeliness of diagnosis, for example, through patient navigation [30], explicit referral pathways, clinical decision support tools, continuous education and training, and developments in novel diagnostics.

The strength of this study lies in its use of data linkage of detailed, deidentified patient records between primary care and hospital datasets, enabling investigations such as this study into the patient journey that begins in primary care and then proceeds to hospital care, including disease diagnosis and treatment [20]. This data linkage enabled this study to investigate the time from primary care to diagnosis and treatment for thoracic cancers. Compared to previous studies using surveys to understand the timeliness from primary care in Australia [44], data linkage between the established datasets may, to some extent, address selection and recall biases as a concern in survey studies. Other strengths include the rich and accurate patient information recorded in our large-scale datasets, which enable further investigations into some of the risk factors for diagnostic delays. In addition, we used the ascertained dates of pathological diagnosis and cancer treatment initiation to construct time intervals in concordance with the Aarhus statement [16].

Regarding the limitations, the linked sample size for analysis was relatively small, given the original sample sizes of the cancer registry dataset. The main reasons include the coverage of available primary care data and the lack of valid encounter records in the primary care datasets, which hindered the construction of DI and DTI. This also precluded more detailed examination of the primary care interval from first presentation to referral. A further limitation is that we did not have access to the general practice clinical history free text field; instead, we had access only to the reason for the encounter. It is quite likely that we missed some relevant visits because the symptoms or signs were recorded in the clinical history field. In an attempt to address this, we made several efforts to identify relevant primary care encounters, including the use of multiple data fields in the medical record (e.g., reasons for visit, blood test results, recorded weight measurements, and radiology request) and consideration of other pulmonary diseases (e.g., chronic obstructive pulmonary disease and asthma) sharing symptoms or signs with thoracic cancers. To avoid potential misattribution of a symptom to the diagnosis of lung cancer, we limited the time window of these encounters to within 1 year of cancer diagnosis, as suggested in previous diagnostic window studies [31–33]. The nature of our linked datasets meant that it was not possible to explore the potential impacts of out-of-pocket costs and public versus private referral pathways on DIs. It was not possible to examine additional patient contributors to overall diagnostic delay that affect symptom appraisal and decisions to seek medical help. We had limited information about patient-level variables such as socioeconomic status, residential remoteness, health literacy, and country of birth. Last, the results may not be generalizable to the whole population of thoracic cancer patients in Victoria/Australia, given the small sample size after linkage and the limited number of hospitals involved.

Notably, the analytic approach of investigating time intervals against the recommended time frames has been widely adopted in research [44–50], particularly for analyzing the proportion of patients who had experienced delays and the risk factors for delays. However, the Optimal Care Pathways recommended maximum intervals were developed using clinical consensus methods [15]. Further evidence is required to show how much outcomes could be improved if patients were diagnosed within these recommended time frames.

In conclusion, this article provides an enriched description of the methodology for the first data linkage between primary care, hospital, and registry datasets in Australia for thoracic cancer research. Through this initiative, we report the time intervals from the first presentation in primary care to thoracic cancer diagnosis and treatment at hospitals, comparing them against recommended health service indicators. We found a large proportion of patients with time intervals exceeding the maximum recommended time frames. These delays may be attributed to comorbidities, particularly respiratory diseases such as pneumonia and respiratory tract infection, which reflects the difficulties in diagnosing thoracic cancers. Policymakers should pay close attention to thoracic cancer timeliness in Australia, especially in addressing diagnostic delays that occur when patients enter the healthcare system from primary care.

## Ethical Approval

Data access, linkage, and the related ethical approval process were carried out through collaborations among the VCCC Alliance, BioGrid Australia, and the University of Melbourne. The protocol for this study received ethical approval from the Melbourne Health Human Research Ethics Committee in 2020 (BioGrid project number: 202005/8).

## Data Availability

Data used in this study are not available to the public, and access may require applications to multiple data custodians, including the VCCC Data Connect, the BioGrid Australia, the University of Melbourne, and the AURORA.
