# Supplementary material for: Diagnostic Delays in Thoracic Cancer Care: A Data-Linkage, Cohort Study between Primary Care, Hospital, and Registry Data
Source: Health Data Sci. 2026 May 4;6:0457. doi: 10.34133/hds.0457 (PMC13136618; doi:10.34133/hds.0457)
Supplement: Supplementary 1 — Tables S1 to S14 [file hds.0457.f1.zip › Supplemental materials_3rd Submission.docx]

Table S1. Details of exposure and outcome variables

| **Variables** | **Source** | |
| --- | --- | --- |
|  | **Primary care datasets (MedicineInsight + Patron)** | **Hospital datasets (AURORA)** |
| **Key variables to build study cohorts** |  |  |
| Patient ID, called Unique Subject Identifier (USI) across datasets | X | X |
| Diagnostic interval (DI), established by the below variables: |  |  |
| Date of 1st primary care encounter (within 12 months before date of diagnosis) | X |  |
| 1^st^ primary care encounter type, classified as: symptom or sign, radiology (CT or X-ray) request, observed weight loss, thrombocytosis | X |  |
| Date of cancer diagnosis |  | X |
| Diagnostic and treatment interval (DTI), established by the below variables: |  |  |
| Date of 1st encounter (same as above) | X |  |
| 1^st^ encounter type (same as above) | X |  |
| Date of 1^st^ cancer treatment (within 12 months after date of diagnosis) |  | X |
| 1^st^ cancer treatment type, classified as: surgery, systemic therapy, radiotherapy, systemic therapy and radiotherapy on the same day |  | X |
| **Demographic characteristics** |  |  |
| Sex: Female, male |  | X |
| Age at diagnosis, established by the below variables: |  |  |
| Date of birth |  | X |
| Date of cancer diagnosis |  | X |
| Year of cancer diagnosis |  | X |
| Ethnicity: White/Caucasian, other |  | X |
| **Environmental & behavioural exposure** |  |  |
| Tobacco use (>=100 lifetime cigarettes): Never, past, current |  | X |
| Marijuana use (>=50 lifetime consumptions): Never, past, current |  | X |
| Asbestos exposure: No, unknown, yes |  | X |
| **Comorbidities**^a^ |  |  |
| Overall: None, other^b^, at least one of the below comorbidities |  | X |
| Cardiovascular diseases^c^: No, yes |  | X |
| Respiratory diseases^d^: No, unknown, yes |  | X |
| Neoplastic comorbidities^e^: No, yes |  | X |
| Renal insufficiency (creatinine clearance lower than 60ml/min): No, yes |  | X |
| Diabetes (the disease treated with oral hypoglycemics or insulin): No, unknown, yes |  | X |
| Alcoholism (daily consumption of 80g for men and 40g for women): No, unknown, yes |  | X |
| **Clinical status** |  |  |
| Histopathological type^f^: NSCLC, other |  | X |
| Stage of lung cancer at diagnosis^g^: 0, I, II, III, IV |  | X |

AURORA, AUstralian Registry and biObank of thoRAcic cancers; CT, computed tomography; NSCLC, non-small-cell lung cancer.

^a^The selection for comorbidities was based on the [Colinet defined comorbidities code](https://meteor.aihw.gov.au/content/432994).

^b^The rest after excluding those with at least one comorbidity and those without any comorbidity.

^c^Presence of one or more of the following: congestive heart failure, ischaemic cardiopathy with or without myocardial infarction, severe valvular cardiopathy, arrhythmia requiring chronic treatment, history of cerebrovascular disease, hypertension and/or peripheral vascular disease.

^d^Presence of one or more of the following: history of tuberculosis, history of pleural effusion or pneumonia, asthma, pulmonary embolism, chronic pulmonary insufficiency as defined by chronic hypoxemia less than 60mmHg and/or chronic obstructive pulmonary disease (COPD) inducing a forced expiratory volume in one second (FEV1) less than 1.5L.

^e^Previous personal history of cancer, excluding basal cell carcinoma of the skin and in situ carcinoma of the cervix.

^f^According to the 2021 WHO Classification of Lung Tumors (5th Edition).

^g^The classification was based on either the 7^th^ or 8^th^ edition of the International Association for the Study of Lung Cancer (IASLC) TNM Classification for Thoracic Cancers, depending on the availability of the information in data; if both are recorded for the same patients, the classification based on the 8^th^ edition was used.

Table S2. Symptoms of lung cancer according to the Australian guidelines [28]

| **Symptoms & signs** | | **Action by general practitioners** |
| --- | --- | --- |
| Massive haemoptysis  Stridor | | Immediate referral to emergency |
| Persistent or unexplained haemoptysis  Signs of superior vena caval obstruction  High clinical suspicion of lung cancer | | Urgent referral to 1) chest computed tomography (CT) scan and 2) specialist |
| Unexplained haemoptysis or any of the following unexplained: persistent symptoms and signs lasting more than 3 weeks (or less than 3 weeks in people with known risk factors^a^): | | Urgent referral to chest X-ray scan |
| • New or changed cough | • Deep vein thrombosis (DVT) |  |
| • Chest and/or shoulder pain | • Abnormal chest signs |  |
| • Shortness of breath | • Finger clubbing |  |
| • Hoarseness | • Cervical and/or supraclavicular lymphadenopathy |  |
| • Weight loss/loss of appetite | • Features suggestive of lung cancer metastasis |  |
| • Persistent or recurrent chest infection | • Pleural effusion |  |
| • Abnormal chest signs | • Thrombocytosis |  |
| • Finger clubbing | • Hoarseness |  |
| • Fatigue |  |  |

^a^(Lifestyle factors) Current or former tobacco smoking; (environmental factors) passive smoking, radon exposure, occupational exposure (e.g. asbestos, diesel exhaust, silica), air pollution; (personal factors) increasing age, family history of lung cancer, chronic lung disease (e.g. chronic obstructive pulmonary disease [COPD], pulmonary fibrosis), personal history of cancer (e.g. lung cancer, head and neck cancer, bladder cancer)

**Table S3. Characteristics between data-linkage patients for analysis and patients ineligible or without data linkage**

| **Characteristics** | **Thoracic cancer** | | | **Non-small-cell lung cancer** | | |
| --- | --- | --- | --- | --- | --- | --- |
|  | **Data-linkage patients for TDI cohort** | **Patients without data linkage** | **P value** | **Data-linkage patients for TDI cohort** | **Patients without data linkage** | **P value** |
| Overall | 249 | 2914 |  | 206 | 2585 |  |
| **Demographic characteristics** | |  |  |  |  |  |
| Sex |  |  | 0.62 |  |  | 0.94 |
| Female | 103 (41%) | 1253 (43%) |  | 89 (43%) | 1110 (43%) |  |
| Male | 146 (59%) | 1661 (57%) |  | 117 (57%) | 1475 (57%) |  |
| Age at diagnosis |  |  | 0.14 |  |  | 0.03 |
| <65 | 95 (38%) | 1213 (42%) |  | 75 (36%) | 1084 (42%) |  |
| >=65 & <75 | 87 (35%) | 1038 (36%) |  | 69 (34%) | 899 (35%) |  |
| >=75 | 67 (27%) | 663 (22%) |  | 62 (30%) | 602 (23%) |  |
| Year of diagnosis |  |  | 0.24 |  |  | 0.19 |
| 2005-2011 | 22 (9%) | 259 (9%) |  | 18 (9%) | 231 (9%) |  |
| 2012-2015 | 108 (43%) | 1185 (41%) |  | 91 (44%) | 1062 (41%) |  |
| 2016-2019 | 104 (42%) | 1194 (41%) |  | 87 (42%) | 1046 (40%) |  |
| 2020-2021 | 15 (6%) | 276 (9%) |  | 10 (5%) | 246 (10%) |  |
| Ethnicity |  |  | 0.01 |  |  | 0.01 |
| White/Caucasian | 226 (91%) | 2473 (85%) |  | 188 (91%) | 2167 (84%) |  |
| Other | 23 (9%) | 440 (15%) |  | 18 (9%) | 417 (16%) |  |
| Missing | 0 | 1 |  | 0 | 1 |  |
| **Environmental & behavioural exposure** | |  |  |  |  |  |
| Tobacco use |  |  | <0.01 |  |  | 0.01 |
| Never | 33 (14%) | 506 (20%) |  | 30 (15%) | 460 (20%) |  |
| Past | 145 (62%) | 1666 (64%) |  | 122 (63%) | 1465 (65%) |  |
| Current | 57 (24%) | 409 (16%) |  | 42 (22%) | 342 (15%) |  |
| Missing | 14 | 333 |  | 12 | 318 |  |
| Marijuana |  |  | 0.01 |  |  | 0.04 |
| Never | 186 (87%) | 1915 (92%) |  | 155 (88%) | 1650 (92%) |  |
| Current or past | 29 (13%) | 164 (8%) |  | 22 (12%) | 140 (8%) |  |
| Missing | 34 | 835 |  | 29 | 795 |  |
| Asbestos |  |  | 0.85 |  |  | 0.97 |
| No | 163 (72%) | 1644 (73%) |  | 138 (75%) | 1445 (75%) |  |
| Yes | 44 (20%) | 437 (20%) |  | 34 (18%) | 356 (18%) |  |
| Unknown | 18 (8%) | 157 (7%) |  | 13 (7%) | 127 (7%) |  |
| Missing | 24 | 676 |  | 21 | 657 |  |
| Alcoholism |  |  | <0.01 |  |  | <0.01 |
| No | 210 (84%) | 2034 (70%) |  | 177 (86%) | 1749 (68%) |  |
| Yes | 16 (6%) | 205 (7%) |  | 8 (4%) | 181 (7%) |  |
| Unknown | 23 (9%) | 672 (23%) |  | 21 (10%) | 652 (25%) |  |
| Missing | 0 | 3 |  | 0 | 3 |  |
| **Comorbidities** |  |  |  |  |  |  |
| Overall |  |  | 0.03 |  |  | 0.03 |
| None | 53 (21%) | 618 (21%) |  | 43 (21%) | 541 (21%) |  |
| At least one comorbidity | 179 (72%) | 1738 (60%) |  | 147 (71%) | 1503 (58%) |  |
| Other^a^ | 17 (7%) | 555 (19%) |  | 16 (8%) | 538 (21%) |  |
| Missing | 0 | 3 |  | 0 | 3 |  |
| Cardiovascular diseases |  |  | <0.01 |  |  | <0.01 |
| No | 141 (57%) | 1942 (67%) |  | 117 (57%) | 1760 (68%) |  |
| Yes | 108 (43%) | 966 (33%) |  | 89 (43%) | 819 (32%) |  |
| Missing | 0 | 6 |  | 0 | 6 |  |
| Respiratory diseases |  |  | 0.48 |  |  | 0.79 |
| No | 135 (54%) | 1437 (49%) |  | 131 (55%) | 1248 (48%) |  |
| Yes | 96 (39%) | 863 (30%) |  | 76 (37%) | 742 (29%) |  |
| Unknown | 18 (7%) | 611 (21%) |  | 17 (8%) | 592 (23%) |  |
| Missing | 0 | 3 |  | 0 | 3 |  |
| Neoplastic comorbidities |  |  | 0.40 |  |  | 0.49 |
| No | 199 (80%) | 2257 (78%) |  | 165 (80%) | 2014 (78%) |  |
| Yes | 50 (20%) | 651 (22%) |  | 41 (20%) | 567 (22%) |  |
| Missing | 0 | 6 |  | 0 | 4 |  |
| Renal insufficiency |  |  | 0.09 |  |  | 0.14 |
| No | 234 (94%) | 2798 (96%) |  | 194 (94%) | 2484 (96%) |  |
| Yes | 15 (6%) | 111 (4%) |  | 12 (6%) | 97 (4%) |  |
| Missing | 0 | 5 |  | 0 | 4 |  |
| Diabetes |  |  | 0.84 |  |  | 0.97 |
| No | 177 (71%) | 1882 (65%) |  | 146 (71%) | 1633 (63%) |  |
| Yes | 49 (20%) | 359 (12%) |  | 39 (19%) | 298 (12%) |  |
| Unknown | 23 (9%) | 669 (23%) |  | 21 (10%) | 650 (25%) |  |
| Missing | 0 | 4 |  | 0 | 4 |  |
| **Clinical status** |  |  |  |  |  |  |
| Histopathological type |  |  | 0.01 |  |  | NA |
| NSCLC | 206 (83%) | 2585 (89%) |  | NA | NA |  |
| Other | 41 (17%) | 319 (11%) |  | NA | NA |  |
| Missing | 2 | 10 |  | NA | NA |  |
| Stage |  |  | <0.01 |  |  | 0.01 |
| I-II | 71 (29%) | 1116 (39%) |  | 64 (31%) | 1058 (41%) |  |
| III-IV | 173 (71%) | 1726 (61%) |  | 141 (69%) | 1509 (59%) |  |
| Missing | 5 | 72 |  | 1 | 18 |  |
| 1^st^ cancer treatment |  |  | 0.04 |  |  | <0.01 |
| Systemic therapy | 60 (24%) | 615 (21%) |  | 37 (18%) | 439 (17%) |  |
| Surgery | 58 (23%) | 1126 (39%) |  | 53 (26%) | 1072 (41%) |  |
| Radiotherapy | 94 (38%) | 843 (29%) |  | 80 (39%) | 764 (30%) |  |
| Systemic therapy+radiotherapy^b^ | 37 (15%) | 330 (11%) |  | 36 (17%) | 310 (12%) |  |

DTI, diagnostic and treatment interval; NSCLC, non-small-cell lung cancer; CT, computed tomography.

^a^The rest after excluding those with at least one comorbidity and those without any comorbidity.

^b^Both systemic therapy and radiotherapy were initiated on the same day.

Table S4. Histopathological types

| **Histopathological type** | **For DI (N=268)** | | **For DTI (N=249)** | |
| --- | --- | --- | --- | --- |
|  | **Frequency** | **Percentage** | **Frequency** | **Percentage** |
| **Non-small-cell lung cancer (NSCLC)** |  |  |  |  |
| Adenocarcinoma | 133 | 49.63% | 128 | 51.41% |
| Squamous cell carcinoma | 64 | 23.88% | 57 | 22.89% |
| Undifferentiated large cell carcinoma | 1 | 0.37% | 1 | 0.40% |
| NSCLC not specified | 18 | 6.72% | 17 | 6.83% |
| Multiple/mixed primary tumour(s) | 4 | 1.49% | 3 | 1.20% |
| **Small-cell lung cancer (SCLC)** |  |  |  |  |
| SCLC | 29 | 10.82% | 27 | 10.84% |
| Multiple/mixed primary tumour(s) | 2 | 0.75% | 2 | 0.80% |
| **Others** |  |  |  |  |
| Other neuroendocrine tumour | 5 | 1.87% | 5 | 2.01% |
| Mesothelioma | 4 | 1.49% | 3 | 1.20% |
| Other (specified) | 4 | 1.49% | 3 | 1.20% |
| Multiple/mixed primary tumour(s) | 1 | 0.37% | 1 | 0.40% |
| **Unknown** | 3 | 1.12% | 2 | 0.80% |

DI: Diagnostic interval; DTI: Diagnostic and treatment interval.

**Table S5. Details of 1^st^ recorded primary care encounters in data-linkage cohorts**

| **1st primary care encounter** | **Cohort for DI (N=268)** | | **Cohort for TDTI (N=249)** | |
| --- | --- | --- | --- | --- |
|  | **Frequency** | **Percentage** | **Frequency** | **Percentage** |
| **Symptom or sign** | **117** | **100%** | **111** | **100%** |
| Pneumonia or infection |  |  |  |  |
| Infection – URTI | 19 | 16.24% | 19 | 17.12% |
| Infection – LRTI | 3 | 2.56% | 3 | 2.70% |
| Infection – Chest | 3 | 2.56% | 3 | 2.70% |
| Pneumonia | 3 | 2.56% | 3 | 2.70% |
| Infection – RTI | 2 | 1.71% | 2 | 1.80% |
| Infection with wheeze | 1 | 0.85% | 1 | 0.90% |
| COPD/COAD | 20 | 17.09% | 17 | 15.32% |
| Pain |  |  |  |  |
| Pain – Chest | 6 | 5.13% | 6 | 5.41% |
| Pain – Shoulder | 3 | 2.56% | 3 | 2.70% |
| Pain – Scapular | 2 | 1.71% | 2 | 1.80% |
| Pain – Throat | 1 | 0.85% | 1 | 0.90% |
| Pain – Pleuritic | 1 | 0.85% | 0 | 0% |
| Cough | 9 | 7.69% | 9 | 8.11% |
| Bronchitis | 8 | 6.84% | 8 | 7.21% |
| Asthma | 7 | 5.98% | 7 | 6.31% |
| Lung lesion | 4 | 3.42% | 3 | 2.70% |
| Radiology | 3 | 2.56% | 3 | 2.70% |
| Rib fracture | 3 | 2.56% | 2 | 1.80% |
| Other | 16 | 13.68% | 16 | 14.41% |
| Multiple relevant symptoms/signs | 3 | 2.56% | 3 | 2.70% |
|  |  |  |  |  |
| **Radiology** | **106** | **100%** | **96** | **100%** |
| X-ray | 70 | 66.04% | 65 | 67.71% |
| CT | 31 | 29.25% | 27 | 28.13% |
| Both on the same date | 5 | 4.72% | 4 | 4.17% |
|  |  |  |  |  |
| **Observed weight loss** | **37** | **100%** | **34** | **100%** |
|  |  |  |  |  |
| **Thrombocytosis** | **8** | **100%** | **8** | **100%** |

DI, diagnostic interval; DTI, diagnostic and treatment interval; URTI, upper respiratory tract infection; LRTI, lower respiratory tract infection; RTI, respiratory tract infection; COPD, chronic obstructive pulmonary disease; COAD, chronic obstructive airways disease; CT, computed tomography.

**Table S6. Patient characteristics in data-linkage cohorts with non-small-cell lung cancer**

| **Characteristics** | **Cohort for diagnostic interval (DI)** | | | **Cohort for diagnostic and treatment interval (DTI)** | | |
| --- | --- | --- | --- | --- | --- | --- |
|  | **Delays (>35 days) (N=165; 75%)** | **No delays (<=35 days) (N=55; 25%)** | **Total**  **(N=220)** | **Delays (>49 days) (N=177; 86%)** | **No delays (<=49 days) (N=29; 14%)** | **Total**  **(N=206)** |
| **Demographic characteristics** | |  |  |  |  |  |
| Sex |  |  |  |  |  |  |
| Female | 71 (74%) | 25 (26%) | 96 | 76 (85%) | 13 (15%) | 89 |
| Male | 94 (76%) | 30 (24%) | 124 | 101 (86%) | 16 (14%) | 117 |
| Age at diagnosis |  |  |  |  |  |  |
| <65 | 51 (67%) | 25 (33%) | 76 | 58 (77%) | 17 (23%) | 75 |
| >=65 & <75 | 57 (77%) | 17 (23%) | 74 | 61 (88%) | 8 (12%) | 69 |
| >=75 | 57 (81%) | 13 (19%) | 70 | 58 (94%) | 4 (6%) | 62 |
| Year of diagnosis |  |  |  |  |  |  |
| 2005-2011 | 13 (68%) | 6 (32%) | 19 | 16 (89%) | 2 (11%) | 18 |
| 2012-2015 | 76 (78%) | 22 (22%) | 98 | 78 (86%) | 13 (14%) | 91 |
| 2016-2019 | 69 (75%) | 22 (25%) | 92 | 76 (87%) | 11 (13%) | 87 |
| 2020-2021 | 7 (64%) | 4 (36%) | 11 | 7 (70%) | 3 (30%) | 10 |
| Ethnicity |  |  |  |  |  |  |
| White | 150 (75%) | 51 (25%) | 201 | 161 (86%) | 27 (14%) | 188 |
| Other | 15 (79%) | 4 (21%) | 19 | 16 (89%) | 2 (11%) | 18 |
| **Environmental & behavioural exposure** | |  |  |  |  |  |
| Tobacco use |  |  |  |  |  |  |
| Never | 23 (72%) | 9 (28%) | 32 | 24 (80%) | 6 (20%) | 30 |
| Past | 97 (73%) | 35 (27%) | 132 | 105 (86%) | 17 (14%) | 122 |
| Current | 33 (75%) | 11 (25%) | 44 | 36 (86%) | 6 (14%) | 42 |
| Missing | NA | NA | 12 | NA | NA | 12 |
| Marijuana |  |  |  |  |  |  |
| Never | 126 (75%) | 42 (25%) | 168 | 135 (87%) | 20 (13%) | 155 |
| Current & past | 12 (55%) | 10 (45%) | 22 | 14 (64%) | 8 (36%) | 22 |
| Missing | NA | NA | 30 | NA | NA | 29 |
| Asbestos |  |  |  |  |  |  |
| No | 111 (76%) | 36 (24%) | 147 | 119 (86%) | 19 (14%) | 138 |
| Yes | 20 (57%) | 15 (43%) | 35 | 26 (76%) | 8 (24%) | 34 |
| Unknown | NA | NA | 16 | NA | NA | 13 |
| Missing | NA | NA | 22 | NA | NA | 21 |
| Alcoholism |  |  |  |  |  |  |
| No | 136 (72%) | 53 (28%) | 189 | 149 (84%) | 28 (16%) | 177 |
| Yes | 7 (78%) | 2 (22%) | 9 | 7 (88%) | 1 (12%) | 8 |
| Unknown | NA | NA | 22 | NA | NA | 21 |
| **Comorbidities** |  |  |  |  |  |  |
| Overall |  |  |  |  |  |  |
| None | 22 (50%) | 22 (50%) | 44 | 29 (67%) | 14 (33%) | 43 |
| At least one comorbidity | 127 (79%) | 33 (21%) | 160 | 132 (90%) | 15 (10%) | 147 |
| Other^b^ | NA | NA | 16 | NA | NA | 16 |
| Cardiovascular diseases |  |  |  |  |  |  |
| No | 86 (69%) | 38 (31%) | 124 | 95 (81%) | 22 (19%) | 117 |
| Yes | 79 (82%) | 17 (18%) | 96 | 82 (92%) | 7 (8%) | 89 |
| Respiratory diseases |  |  |  |  |  |  |
| No | 76 (65%) | 41 (35%) | 117 | 90 (80%) | 23 (20%) | 113 |
| Yes | 72 (84%) | 14 (16%) | 86 | 70 (92%) | 6 (8%) | 76 |
| Unknown | NA | NA | 17 | NA | NA | 17 |
| Neoplastic comorbidities |  |  |  |  |  |  |
| No | 128 (74%) | 44 (26%) | 172 | 139 (84%) | 26 (16%) | 165 |
| Yes | 37 (77%) | 11 (23%) | 48 | 38 (93%) | 3 (7%) | 41 |
| Renal insufficiency |  |  |  |  |  |  |
| No | 155 (75%) | 53 (25%) | 208 | 167 (86%) | 27 (14%) | 194 |
| Yes | 10 (83%) | 2 (17%) | 12 | 10 (83%) | 2 (17%) | 12 |
| Diabetes |  |  |  |  |  |  |
| No | 107 (68%) | 50 (32%) | 157 | 120 (82%) | 26 (18%) | 146 |
| Yes | 36 (88%) | 5 (12%) | 41 | 36 (92%) | 3 (8%) | 39 |
| Unknown | NA | NA | 22 | NA | NA | 21 |
| **Clinical status** |  |  |  |  |  |  |
| Stage |  |  |  |  |  |  |
| I-II | 63 (93%) | 5 (7%) | 68 | 61 (95%) | 3 (5%) | 64 |
| III-IV | 101 (67%) | 50 (33%) | 151 | 115 (82%) | 26 (18%) | 141 |
| Missing | NA | NA | 1 | NA | NA | 1 |
| 1^st^ primary care encounter type |  |  |  |  |  |  |
| Symptom or sign | 76 (77%) | 23 (23%) | 99 | 82 (85%) | 14 (15%) | 96 |
| Radiology request | 55 (65%) | 30 (35%) | 85 | 63 (82%) | 14 (18%) | 77 |
| Observed weight loss | 27 (93%) | 2 (7%) | 29 | 25 (96%) | 1 (4%) | 26 |
| Thrombocytosis | 7 (100%) | 0 | 7 | 7 (100%) | 0 | 7 |
| 1^st^ cancer treatment |  |  |  |  |  |  |
| Systemic therapy | NA | NA | 37 | 30 (81%) | 7 (19%) | 37 |
| Surgery | NA | NA | 53 | 49 (92%) | 4 (8%) | 53 |
| Radiotherapy | NA | NA | 80 | 64 (80%) | 16 (20%) | 80 |
| Systemic therapy+radiotherapy^b^ | NA | NA | 36 | 34 (94%) | 2 (6%) | 36 |
| Missing | NA | NA | 14 | NA | NA | 0 |

NA: Not applicable.

^a^The rest after excluding those with at least one comorbidity and those without any comorbidity.

^b^Both systemic therapy and radiotherapy were initiated on the same day.

Table S7. Associations between characteristics and diagnostic delay (diagnostic interval >35 days) in patients with any thoracic cancer

| **Characteristics** | **Odds ratio**  **(Model 1)** | **Odds ratio (Model 2)** | **Odds ratio (Model 3)** | **Odds ratio (Model 4)** | **Odds ratio (Model 5)** |
| --- | --- | --- | --- | --- | --- |
| **Demographic characteristics** |  |  |  |  |  |
| Sex: Male vs. Female | 1.08 (0.61-1.91) | 1.04 (0.58-1.85) | 0.99 (0.56-1.77) | 0.99 (0.56-1.78) | 0.97 (0.53-1.75) |
| Age at diagnosis |  |  |  |  |  |
| <65 | 0.61 (0.32-1.18) | 0.61 (0.32-1.18) | 0.60 (0.31-1.16) | 0.60 (0.31-1.16) | 0.58 (0.29-1.13) |
| >=65 & <75 | 1.00 | 1.00 | 1.00 | 1.00 | 1.00 |
| >=75 | 1.37 (0.63-2.97) | 1.37 (0.63-2.97) | 1.40 (0.64-3.07) | 1.40 (0.64-3.07) | 1.37 (0.62-3.05) |
| Year of diagnosis |  |  |  |  |  |
| 2005-2011 | 0.83 (0.31-2.19) | 0.90 (0.34-2.41) | 0.84 (0.31-2.27) | NA | 1.02 (0.37-2.78) |
| 2012-2015 | 1.17 (0.63-2.17) | 1.22 (0.65-2.29) | 1.23 (0.65-2.31) | NA | 1.32 (0.69-2.52) |
| 2016-2019 | 1.00 | 1.00 | 1.00 | NA | 1.00 |
| 2020-2021 | 0.96 (0.29-3.24) | 0.94 (0.28-3.17) | 0.88 (0.26-3.01) | NA | 0.96 (0.27-3.33) |
| Ethnicity: White/Caucasian vs. Other | 0.97 (0.37-2.55) | 1.14 (0.43-3.06) | 1.12 (0.42-3.04) | 1.12 (0.42-3.04) | 1.12 (0.41-3.09) |
| **Environmental & behavioural exposure** |  |  |  |  |  |
| Tobacco use |  |  |  |  |  |
| Never | 1.00 | 1.00 | 1.00 | 1.00 | 1.00 |
| Past | 0.96 (0.41-2.21) | 0.78 (0.32-1.90) | 0.77 (0.31-1.87) | 0.76 (0.31-1.89) | 0.92 (0.36-2.36) |
| Current | 1.41 (0.53-3.79) | 1.41 (0.51-3.87) | 1.31 (0.47-3.65) | 1.30 (0.46-3.71) | 1.65 (0.56-4.83) |
| Marijuana: Current or Past vs. Never | 0.58 (0.26-1.30) | 0.71 (0.30-1.68) | 0.61 (0.25-1.51) | 0.61 (0.25-1.52) | 0.62 (0.25-1.52) |
| Asbestos: Yes vs. No | 0.48 (0.24-0.95)* | 0.45 (0.22-0.92)* | 0.43 (0.21-0.89)* | 0.43 (0.20-0.89)* | 0.43 (0.20-0.89)* |
| Alcoholism: Yes vs. No | 1.94 (0.55-6.89) | 2.26 (0.62-8.25) | 1.97 (0.52-7.43) | 1.96 (0.52-7.44) | 1.97 (0.52-7.50) |
| **Comorbidities** |  |  |  |  |  |
| Any comorbidities: At least one vs. None | 2.91 (1.53-5.53)* | 2.59 (1.29-5.18)* | 2.58 (1.28-5.19)* | 2.59 (1.28-5.22)* | 2.49 (1.23-5.05)* |
| Cardiovascular diseases: Yes vs. No | 1.67 (0.93-3.01) | 1.42 (0.76-2.67) | 1.40 (0.75-2.63) | 1.41 (0.75-2.67) | 1.81 (0.94-3.48) |
| Respiratory diseases: Yes vs. No | 2.91 (1.54-5.49)* | 2.74 (1.44-5.22)* | 2.65 (1.39-5.06)* | 2.67 (1.39-5.12)* | 2.51 (1.30-4.83)* |
| Neoplastic comorbidities: Yes vs. No | 1.14 (0.57-2.28) | 0.97 (0.48-1.99) | 0.96 (0.47-1.97) | 0.96 (0.47-1.97) | 1.14 (0.55-2.35) |
| Renal insufficiency: Yes vs. No | 1.24 (0.34-4.55) | 1.10 (0.30-4.07) | 1.10 (0.30-4.11) | 1.11 (0.30-4.13) | 1.20 (0.32-4.53) |
| Diabetes: Yes vs. No | 2.14 (0.95-4.85) | 1.92 (0.84-4.41) | 1.94 (0.84-4.48) | 1.93 (0.84-4.48) | 1.93 (0.84-4.48) |
| **Clinical status** |  |  |  |  |  |
| Histopathological type: Other vs. NSCLC | 1.54 (0.68-3.51) | 1.66 (0.72-3.81) | NA | 1.66 (0.72-3.81) | 1.84 (0.79-4.28) |
| Stage: I-II vs. III-IV | 5.24 (2.15-12.76)* | 4.93 (2.01-12.10)* | 5.19 (2.10-12.82)* | 5.23 (2.11-12.96)* | 3.89 (1.55-9.77)* |
| 1^st^ primary care encounter type |  |  |  |  |  |
| Symptom or sign | 1.00 | 1.00 | 1.00 | 1.00 | 1.00 |
| Radiology request (CT or X-ray) | 0.64 (0.35-1.15) | 0.59 (0.32-1.08) | 0.57 (0.31-1.05) | 0.57 (0.31-1.05) | 0.49 (0.26-0.91)* |
| Observed weight loss | 5.25 (1.19-23.26)* | 4.94 (1.11-22.06)* | 4.78 (1.07-21.46)* | 4.78 (1.07-21.46)* | 4.41 (0.97-20.04) |
| Thrombocytosis | NA | NA | NA | NA | NA |

Data in parentheses are 95% confidence intervals.

NSCLC, non-small-cell lung cancer; CT, computed tomography; NA, not applicable.

^*^Statistically significant, as indicated by the p value lower than 0.05.

Adjustment: (model 1) no adjustment; (model 2) sex, age; (model 3) sex, age, histopathology; (model 4) sex, age, histopathology, year of diagnosis; (model 5) sex, age, histopathology, year of diagnosis, ethnicity, hospital.

Table S8. Associations between characteristics and diagnostic delay (diagnostic interval >35 days) in patients with non-small-cell lung cancer

| **Characteristics** | **Odds ratio**  **(Model 1)** | **Odds ratio**  **(Model 2)** | **Odds ratio**  **(Model 3)** | **Odds ratio**  **(Model 4)** |
| --- | --- | --- | --- | --- |
| **Demographic characteristics** |  |  |  |  |
| Sex: Male vs. Female | 1.10 (0.60-2.04) | 1.08 (0.58-2.00) | 1.07 (0.58-1.99) | 1.04 (0.55-1.97) |
| Age at diagnosis |  |  |  |  |
| <65 | 0.61 (0.30-1.25) | 0.61 (0.30-1.26) | 0.61 (0.30-1.26) | 0.57 (0.27-1.20) |
| >=65 & <75 | 1.00 | 1.00 | 1.00 | 1.00 |
| >=75 | 1.31 (0.58-2.94) | 1.31 (0.58-2.94) | 1.31 (0.58-2.94) | 1.31 (0.57-2.99) |
| Year of diagnosis |  |  |  |  |
| 2005-2011 | 0.72 (0.25-2.12) | 0.77 (0.26-2.28) | NA | 0.96 (0.32-2.87) |
| 2012-2015 | 1.15 (0.59-2.25) | 1.20 (0.61-2.38) | NA | 1.31 (0.65-2.63) |
| 2016-2019 | 1.00 | 1.00 | NA | 1.00 |
| 2020-2021 | 0.58 (0.16-2.17) | 0.56 (0.15-2.10) | NA | 0.57 (0.14-2.24) |
| Ethnicity: White/Caucasian vs. Other | 1.28 (0.40-4.02) | 1.57 (0.48-5.14) | 1.59 (0.48-5.20) | 1.60 (0.48-5.34) |
| **Environmental & behavioural exposure** |  |  |  |  |
| Tobacco use |  |  |  |  |
| Never | 1.00 | 1.00 | 1.00 | 1.00 |
| Past | 1.08 (0.46-2.57) | 0.93 (0.37-2.32) | 0.91 (0.36-2.29) | 1.19 (0.45-3.13) |
| Current | 1.17 (0.42-3.29) | 1.20 (0.41-3.45) | 1.16 (0.40-3.39) | 1.58 (0.52-4.82) |
| Marijuana: Current or Past vs. Never | 0.40 (0.16-0.99)* | 0.47 (0.17-1.28) | 0.48 (0.18-1.31) | 0.49 (0.18-1.35) |
| Asbestos: Yes vs. No | 0.43 (0.20-0.93)* | 0.42 (0.19-0.93)* | 0.41 (0.18-0.92)* | 0.41 (0.18-0.93)* |
| Alcoholism: Yes vs. No | 1.36 (0.27-6.78) | 1.58 (0.30-8.20) | 1.58 (0.31-8.21) | 1.66 (0.32-8.64) |
| **Comorbidities** |  |  |  |  |
| Any comorbidities: At least one vs. None | 3.85 (1.90-7.78)* | 3.67 (1.71-7.89)* | 3.76 (1.74-8.12)* | 3.72 (1.70-8.12)* |
| Cardiovascular diseases: Yes vs. No | 2.05 (1.07-3.93)* | 1.86 (0.94-3.69) | 1.91 (0.96-3.83) | 2.51 (1.23-5.14)* |
| Respiratory diseases: Yes vs. No | 2.77 (1.40-5.51)* | 2.63 (1.32-5.27)* | 2.62 (1.30-5.27)* | 2.47 (1.22-5.00)* |
| Neoplastic comorbidities: Yes vs. No | 1.16 (0.54-2.46) | 1.01 (0.46-2.20) | 1.01 (0.46-2.21) | 1.27 (0.58-2.80) |
| Renal insufficiency: Yes vs. No | 1.71 (0.36-8.05) | 1.57 (0.33-7.46) | 1.56 (0.33-7.41) | 1.80 (0.38-8.60) |
| Diabetes: Yes vs. No | 3.36 (1.25-9.09)* | 3.05 (1.11-8.35)* | 3.03 (1.11-8.31)* | 3.03 (1.10-8.30)* |
| **Clinical status** |  |  |  |  |
| Stage: I-II vs. III-IV | 6.24 (2.36-16.48)* | 5.91 (2.22-15.74)* | 6.00 (2.25-16.02)* | 4.51 (1.66-12.25)* |
| 1^st^ primary care encounter type |  |  |  |  |
| Symptom or sign | 1.00 | 1.00 | 1.00 | 1.00 |
| Radiology request (CT or X-ray) | 0.55 (0.29-1.06)* | 0.50 (0.26-0.97)* | 0.50 (0.26-0.97)* | 0.41 (0.21-0.82)* |
| Observed weight loss | 4.09 (0.90-18.50) | 3.81 (0.83-17.45) | 3.82 (0.83-17.51) | 3.54 (0.76-16.51) |
| Thrombocytosis | NA | NA | NA | NA |

Data in parentheses are 95% confidence intervals.

CT, computed tomography; NA, not applicable.

^*^Statistically significant, as indicated by the p value lower than 0.05.

Adjustment: (model 1) no adjustment; (model 2) sex, age; (model 3) sex, age, year of diagnosis; (model 4) sex, age, year of diagnosis, ethnicity, hospital.

Table S9. Associations between characteristics and diagnostic and treatment delay (diagnostic and treatment interval >49 days) in patients with any thoracic cancer

| **Characteristics** | **Odds ratio**  **(Model 1)** | **Odds ratio**  **(Model 2)** | **Odds ratio**  **(Model 3)** | **Odds ratio**  **(Model 4)** | **Odds ratio**  **(Model 5)** |
| --- | --- | --- | --- | --- | --- |
| **Demographic characteristics** |  |  |  |  |  |
| Sex: Male vs. Female | 1.01 (0.50-2.08) | 0.95 (0.46-1.97) | 0.94 (0.45-1.96) | 0.92 (0.44-1.92) | 0.91 (0.43-1.91) |
| Age at diagnosis |  |  |  |  |  |
| <65 | 0.60 (0.27-1.31) | 0.60 (0.27-1.31) | 0.59 (0.27-1.30) | 0.59 (0.27-1.29) | 0.58 (0.26-1.28) |
| >=65 & <75 | 1.00 | 1.00 | 1.00 | 1.00 | 1.00 |
| >=75 | 2.52 (0.77-8.20) | 2.52 (0.77-8.21) | 2.47 (0.75-8.10) | 2.48 (0.75-8.15) | 2.44 (0.73-8.08) |
| Year of diagnosis |  |  |  |  |  |
| 2005-2011 | 1.69 (0.36-7.97) | 2.00 (0.41-9.60) | 2.03 (0.42-9.90) | NA | 2.34 (0.47-11.54) |
| 2012-2015 | 0.97 (0.45-2.08) | 1.03 (0.47-2.24) | 1.00 (0.46-2.19) | NA | 1.05 (0.48-2.31) |
| 2016-2019 | 1.00 | 1.00 | 1.00 | NA | 1.00 |
| 2020-2021 | 0.67 (0.17-2.68) | 0.62 (0.15-2.48) | 0.63 (0.15-2.55) | NA | 0.67 (0.16-2.74) |
| Ethnicity: White/Caucasian vs. Other | 0.78 (0.25-2.45) | 1.00 (0.31-3.24) | 1.01 (0.31-3.27) | 1.02 (0.31-3.32) | 1.04 (0.32-3.39) |
| **Environmental & behavioural exposure** |  |  |  |  |  |
| Tobacco use |  |  |  |  |  |
| Never | 1.00 | 1.00 | 1.00 | 1.00 | 1.00 |
| Past | 1.24 (0.46-3.36) | 1.00 (0.34-2.90) | 1.02 (0.35-2.99) | 0.95 (0.32-2.86) | 1.07 (0.35-3.32) |
| Current | 1.59 (0.48-5.20) | 1.59 (0.47-5.39) | 1.60 (0.47-5.48) | 1.46 (0.41-5.18) | 1.72 (0.47-6.26) |
| Marijuana: Current or Past vs. Never | 0.43 (0.17-1.06) | 0.61 (0.22-1.65) | 0.56 (0.20-1.55) | 0.57 (0.21-1.60) | 0.57 (0.20-1.59) |
| Asbestos: Yes vs. No | 0.47 (0.21-1.06) | 0.47 (0.20-1.11) | 0.46 (0.19-1.09) | 0.45 (0.19-1.07) | 0.44 (0.19-1.06) |
| Alcoholism: Yes vs. No | 1.31 (0.28-6.01) | 1.62 (0.34-7.77) | 1.76 (0.35-8.79) | 1.63 (0.33-8.14) | 1.63 (0.33-8.15) |
| **Comorbidities** |  |  |  |  |  |
| Any comorbidities: At least one vs. None | 2.70 (1.26-5.78)* | 2.09 (0.91-4.81) | 2.08 (0.90-4.80) | 2.11 (0.91-4.88) | 2.03 (0.87-4.71) |
| Cardiovascular diseases: Yes vs. No | 1.90 (0.89-4.06) | 1.45 (0.64-3.29) | 1.47 (0.65-3.33) | 1.56 (0.68-3.57) | 1.86 (0.80-4.34) |
| Respiratory diseases: Yes vs. No | 2.75 (1.19-6.36)* | 2.45 (1.05-5.74)* | 2.41 (1.03-5.66)* | 2.35 (1.00-5.54) | 2.21 (0.93-5.24) |
| Neoplastic comorbidities: Yes vs. No | 1.66 (0.61-4.52) | 1.33 (0.47-3.71) | 1.32 (0.47-3.68) | 1.31 (0.47-3.66) | 1.44 (0.51-4.06) |
| Renal insufficiency: Yes vs. No | 0.66 (0.18-2.45) | 0.51 (0.13-1.98) | 0.52 (0.14-2.01) | 0.49 (0.13-1.91) | 0.50 (0.13-1.96) |
| Diabetes: Yes vs. No | 1.40 (0.55-3.60) | 1.13 (0.43-3.00) | 1.13 (0.42-3.02) | 1.10 (0.41-2.97) | 1.10 (0.41-2.97) |
| **Clinical status** |  |  |  |  |  |
| Histopathological type: Other vs. NSCLC | 0.80 (0.32-1.96) | 0.86 (0.35-2.15) | NA | 0.89 (0.35-2.22) | 0.95 (0.37-2.38) |
| Stage: I-II vs. III-IV | 2.88 (1.07-7.74)* | 2.49 (0.91-6.81) | 2.51 (0.91-6.94) | 2.61 (0.93-7.27) | 2.14 (0.74-6.18) |
| 1^st^ primary care encounter type |  |  |  |  |  |
| Symptom or sign | 1.00 | 1.00 | 1.00 | 1.00 | 1.00 |
| Radiology request (CT or X-ray) | 0.68 (0.33-1.42) | 0.62 (0.30-1.31) | 0.63 (0.30-1.35) | 0.63 (0.30-1.34) | 0.58 (0.27-1.24) |
| Observed weight loss | 5.56 (0.71-43.55) | 5.18 (0.65-41.05) | 5.06 (0.64-40.21) | 5.01 (0.63-39.81) | 4.66 (0.58-37.29) |
| Thrombocytosis | NA | NA | NA | NA | NA |
| 1^st^ cancer treatment |  |  |  |  |  |
| Systemic therapy | 1.00 | 1.00 | 1.00 | 1.00 | 1.00 |
| Surgery | 1.95 (0.67-5.66) | 1.64 (0.55-4.86) | 1.69 (0.54-5.23) | 1.65 (0.53-5.13) | 1.11 (0.33-3.76) |
| Radiotherapy | 1.02 (0.44-2.35) | 0.77 (0.32-1.86) | 0.77 (0.31-1.90) | 0.75 (0.30-1.87) | 0.74 (2.29-1.85) |
| Systemic therapy+radiotherapy^a^ | 3.93 (0.82-18.84) | 3.53 (0.73-17.17) | 3.64 (0.72-18.40) | 3.43 (0.67-17.64) | 3.42 (0.66-17.61) |

Data in parentheses are 95% confidence intervals.

NSCLC, non-small-cell lung cancer; CT, computed tomography; NA, not applicable.

^*^Statistically significant, as indicated by the p value lower than 0.05.

Adjustment: (model 1) no adjustment; (model 2) sex, age; (model 3) sex, age, histopathology; (model 4) sex, age, histopathology, year of diagnosis; (model 5) sex, age, histopathology, year of diagnosis, ethnicity, hospital.

^a^Both systemic therapy and radiotherapy were initiated on the same day.

Table S10. Associations between characteristics and diagnostic and treatment delay (diagnostic and treatment interval >49 days) in patients with non-small-cell lung cancer

| **Characteristics** | **Odds ratio**  **(Model 1)** | **Odds ratio**  **(Model 2)** | **Odds ratio**  **(Model 3)** | **Odds ratio**  **(Model 4)** |
| --- | --- | --- | --- | --- |
| **Demographic characteristics** |  |  |  |  |
| Sex: Male vs. Female | 1.08 (0.49-2.38) | 1.01 (0.45-2.25) | 0.98 (0.44-2.21) | 0.97 (0.43-2.21) |
| Age at diagnosis |  |  |  |  |
| <65 | 0.45 (0.18-1.12) | 0.45 (0.18-1.12) | 0.45 (0.18-1.11) | 0.42 (0.17-1.07) |
| >=65 & <75 | 1.00 | 1.00 | 1.00 | 1.00 |
| >=75 | 1.90 (0.54-6.66) | 1.90 (0.54-6.66) | 1.91 (0.55-6.71) | 1.97 (0.55-6.99) |
| Year of diagnosis |  |  |  |  |
| 2005-2011 | 1.16 (0.23-5.74) | 1.28 (0.25-6.48) | NA | 1.61 (0.32-8.23) |
| 2012-2015 | 0.87 (0.37-2.06) | 0.94 (0.39-2.27) | NA | 1.03 (0.42-2.54) |
| 2016-2019 | 1.00 | 1.00 | NA | 1.00 |
| 2020-2021 | 0.34 (0.08-1.50) | 0.29 (0.06-1.35) | NA | 0.29 (0.06-1.40) |
| Ethnicity: White/Caucasian vs. Other | 1.34 (0.29-6.17) | 1.97 (0.40-9.66) | 2.05 (0.41-10.12) | 2.09 (0.42-10.41) |
| **Environmental & behavioural exposure** |  |  |  |  |
| Tobacco use |  |  |  |  |
| Never | 1.00 | 1.00 | 1.00 | 1.00 |
| Past | 1.54 (0.55-4.33) | 1.22 (0.40-3.72) | 1.12 (0.36-3.52) | 1.45 (0.45-4.67) |
| Current | 1.50 (0.43-5.20) | 1.53 (0.42-5.57) | 1.39 (0.37-5.20) | 1.87 (0.48-7.27) |
| Marijuana: Current or Past vs. Never | 0.26 (0.10-0.70)* | 0.35 (0.12-1.05) | 0.36 (0.12-1.10) | 0.37 (0.12-1.13) |
| Asbestos: Yes vs. No | 0.52 (0.21-1.31) | 0.52 (0.19-1.39) | 0.50 (0.19-1.35) | 0.50 (0.19-1.37) |
| Alcoholism: Yes vs. No | 1.32 (0.16-11.11) | 1.69 (0.19-15.20) | 1.70 (0.19-15.09) | 1.81 (0.20-16.21) |
| **Comorbidities** |  |  |  |  |
| Any comorbidities: At least one vs. None | 4.25 (1.85-9.76)* | 3.46 (1.39-8.60)* | 3.62 (1.44-9.09)* | 3.61 (1.42-9.16)* |
| Cardiovascular diseases | 2.71 (1.10-6.67)* | 2.17 (0.84-5.60) | 2.31 (0.88-6.04) | 2.93 (1.10-7.80)* |
| Respiratory diseases: Yes vs. No | 2.98 (1.15-7.72)* | 2.69 (1.02-7.05)* | 2.61 (0.99-6.87) | 2.47 (0.93-6.56) |
| Neoplastic comorbidities: Yes vs. No | 2.37 (0.68-8.25) | 1.94 (0.54-6.91) | 1.96 (0.55-7.01) | 2.45 (0.68-8.83) |
| Renal insufficiency: Yes vs. No | 0.81 (0.17-3.89) | 0.65 (0.13-3.24) | 0.62 (0.12-3.11) | 0.71 (0.14-3.58) |
| Diabetes: Yes vs. No | 2.60 (0.74-9.09) | 2.04 (0.56-7.35) | 2.01 (0.56-7.28) | 2.01 (0.55-7.26) |
| **Clinical status** |  |  |  |  |
| Stage: I-II vs. III-IV | 4.60 (1.34-15.80)* | 3.98 (1.14-13.87)* | 4.09 (1.17-14.29)* | 2.97 (0.83-10.58) |
| 1^st^ primary care encounter type |  |  |  |  |
| Symptom or sign | 1.00 | 1.00 | 1.00 | 1.00 |
| Radiology request (CT or X-ray) | 0.77 (0.34-1.73) | 0.67 (0.29-1.53) | 0.64 (0.28-1.49) | 0.55 (0.23-1.30) |
| Observed weight loss | 4.27 (0.53-34.08) | 3.93 (0.48-31.99) | 3.89 (0.48-31.65) | 3.80 (0.45-31.93) |
| Thrombocytosis | NA | NA | NA | NA |
| 1^st^ cancer treatment |  |  |  |  |
| Systemic therapy | 1.00 | 1.00 | 1.00 | 1.00 |
| Surgery | 2.86 (0.77-10.59) | 2.33 (0.61-8.87) | 2.30 (0.60-8.78) | 1.20 (0.30-4.80) |
| Radiotherapy | 0.93 (0.35-2.51) | 0.63 (0.22-1.81) | 0.63 (0.22-1.80) | 0.64 (0.22-1.88) |
| Systemic therapy+radiotherapy^a^ | 3.97 (0.76-20.58) | 3.45 (0.65-18.32) | 3.33 (0.62-17.97) | 3.28 (0.61-17.76) |

Data in parentheses are 95% confidence intervals.

CT, computed tomography; NA, not applicable.

^*^Statistically significant, as indicated by the p value lower than 0.05.

Adjustment: (model 1) no adjustment; (model 2) sex, age; (model 3) sex, age, year of diagnosis; (model 4) sex, age, year of diagnosis, ethnicity, hospital.

^a^Both systemic therapy and radiotherapy were initiated on the same day.

Table S11. Associations between characteristics and diagnostic delay (diagnostic interval >35 days) in patients with any thoracic cancer (diagnosed in 2011-2019)

| **Characteristics** | **Odds ratio (Model 1)** | **Odds ratio (Model 2)** | **Odds ratio (Model 3)** | **Odds ratio (Model 4)** | **Odds ratio (Model 5)** |
| --- | --- | --- | --- | --- | --- |
| **Demographic characteristics** |  |  |  |  |  |
| Sex: Male vs. Female | 1.00 (0.54-1.84) | 0.96 (0.52-1.79) | 0.91 (0.49-1.71) | 0.90 (0.48-1.68) | 0.85 (0.45-1.62) |
| Age at diagnosis |  |  |  |  |  |
| <65 | 0.69 (0.34-1.38) | 0.69 (0.34-1.38) | 0.68 (0.33-1.36) | 0.68 (0.34-1.38) | 0.65 (0.32-1.33) |
| >=65 & <75 | 1.00 | 1.00 | 1.00 | 1.00 | 1.00 |
| >=75 | 1.38 (0.62-3.11) | 1.38 (0.62-3.11) | 1.40 (0.62-3.17) | 1.42 (0.62-3.23) | 1.41 (0.61-3.27) |
| Year of diagnosis |  |  |  |  |  |
| 2011-2013 | 1.20 (0.58-2.49) | 1.23 (0.58-2.57) | 1.22 (0.58-2.57) | NA | 1.26 (0.59-2.69) |
| 2014-2016 | 1.72 (0.83-3.57) | 1.83 (0.87-3.82) | 1.88 (0.89-3.98) | NA | 1.75 (0.81-3.76) |
| 2017-2019 | 1.00 | 1.00 | 1.00 | NA | 1.00 |
| Ethnicity: White/Caucasian vs. Other | 1.00 (0.35-2.85) | 1.15 (0.40-3.35) | 1.10 (0.37-3.25) | 1.10 (0.37-3.24) | 1.07 (0.35-3.22) |
| **Environmental & behavioural exposure** |  |  |  |  |  |
| Tobacco use |  |  |  |  |  |
| Never | 1.00 | 1.00 | 1.00 | 1.00 | 1.00 |
| Past | 0.72 (0.27-1.89) | 0.58 (0.21-1.64) | 0.60 (0.21-1.69) | 0.58 (0.21-1.66) | 0.70 (0.24-2.07) |
| Current | 0.94 (0.31-2.81) | 0.93 (0.30-2.87) | 0.90 (0.29-2.81) | 0.85 (0.27-2.68) | 1.07 (0.33-3.50) |
| Marijuana: Current or Past vs. Never | 0.51 (0.23-1.17) | 0.61 (0.25-1.46) | 0.52 (0.20-1.30) | 0.53 (0.21-1.33) | 0.53 (0.21-1.33) |
| Asbestos: Yes vs. No | 0.43 (0.21-0.87)* | 0.41 (0.20-0.87)* | 0.39 (0.18-0.83)* | 0.37 (0.18-0.80)* | 0.38 (0.18-0.80)* |
| Alcoholism: Yes vs. No | 1.62 (0.45-5.85) | 1.95 (0.52-7.28) | 1.62 (0.41-6.32) | 1.50 (0.38-5.95) | 1.51 (0.38-5.98) |
| **Comorbidities** |  |  |  |  |  |
| Any comorbidities: At least one vs. None | 2.38 (1.19-4.75)* | 2.08 (0.99-4.37) | 2.09 (0.99-4.42) | 2.10 (0.99-4.45) | 2.01 (0.94-4.29) |
| Cardiovascular diseases: Yes vs. No | 1.55 (0.83-2.89) | 1.33 (0.68-2.59) | 1.32 (0.67-2.58) | 1.38 (0.70-2.72) | 1.75 (0.87-3.51) |
| Respiratory diseases: Yes vs. No | 2.67 (1.37-5.19)* | 2.53 (1.29-4.95)* | 2.48 (1.26-4.88)* | 2.43 (1.23-4.80)* | 2.26 (1.14-4.49)* |
| Neoplastic comorbidities: Yes vs. No | 1.02 (0.51-2.08) | 0.87 (0.42-1.81) | 0.85 (0.41-1.79) | 0.86 (0.41-1.80) | 1.03 (0.49-2.19) |
| Renal insufficiency: Yes vs. No | 0.98 (0.26-3.69) | 0.86 (0.23-3.30) | 0.85 (0.22-3.29) | 0.85 (0.22-3.29) | 0.94 (0.24-3.67) |
| Diabetes: Yes vs. No | 2.46 (0.98-6.19) | 2.28 (0.90-5.79) | 2.36 (0.92-6.05) | 2.35 (0.92-6.03) | 2.35 (0.92-6.03) |
| **Clinical status** |  |  |  |  |  |
| Histopathological type: Other vs. NSCLC | 1.44 (0.60-3.48) | 1.55 (0.64-3.77) | NA | 1.55 (0.64-3.79) | 1.73 (0.70-4.29) |
| Stage: I-II vs. III-IV | 4.91 (2.00-12.08)* | 4.67 (1.88-11.58)* | 4.75 (1.90-11.86)* | 4.98 (1.98-12.55)* | 3.66 (1.43-9.38)* |
| 1^st^ primary care encounter type |  |  |  |  |  |
| Symptom or sign | 1.00 | 1.00 | 1.00 | 1.00 | 1.00 |
| Radiology request (CT or X-ray) | 0.58 (0.31-1.10) | 0.55 (0.29-1.04) | 0.53 (0.28-1.01) | 0.53 (0.28-1.03) | 0.45 (0.23-0.88)* |
| Observed weight loss | 4.24 (0.94-19.07) | 4.09 (0.90-18.50) | 4.03 (0.89-18.36) | 4.06 (0.89-18.48) | 3.73 (0.81-17.14) |
| Thrombocytosis | NA | NA | NA | NA | NA |

Data in parentheses are 95% confidence intervals.

NSCLC, non-small-cell lung cancer; SCLC, small-cell lung cancer; CT, computed tomography; NA, not applicable.

^*^Statistically significant, as indicated by the p value lower than 0.05.

Adjustment: (model 1) no adjustment; (model 2) sex, age; (model 3) sex, age, histopathology; (model 4) sex, age, histopathology, year of diagnosis; (model 5) sex, age, histopathology, year of diagnosis, ethnicity, hospital.

Table S12. Associations between characteristics and diagnostic and treatment delay (diagnostic and treatment interval >49 days) in patients with any thoracic cancer (diagnosed in 2011-2019)

| **Characteristics** | **Odds ratio**  **(Model 1)** | **Odds ratio**  **(Model 2)** | **Odds ratio**  **(Model 3)** | **Odds ratio**  **(Model 4)** | **Odds ratio**  **(Model 5)** |
| --- | --- | --- | --- | --- | --- |
| **Demographic characteristics** |  |  |  |  |  |
| Sex: Male vs. Female | 1.02 (0.48-2.19) | 0.96 (0.44-2.08) | 0.99 (0.45-2.16) | 0.97 (0.44-2.13) | 0.96 (0.43-2.14) |
| Age at diagnosis |  |  |  |  |  |
| <65 | 0.66 (0.29-1.51) | 0.66 (0.29-1.51) | 0.65 (0.28-1.49) | 0.65 (0.28-1.50) | 0.65 (0.28-1.49) |
| >=65 & <75 | 1.00 | 1.00 | 1.00 | 1.00 | 1.00 |
| >=75 | 2.34 (0.71-7.75) | 2.34 (0.71-7.75) | 2.19 (0.65-7.34) | 2.21 (0.66-7.42) | 2.23 (0.66-7.55) |
| Year of diagnosis |  |  |  |  |  |
| 2011-2013 | 1.08 (0.43-2.71) | 1.11 (0.43-2.84) | 1.09 (0.42-2.79) | NA | 1.12 (0.43-2.88) |
| 2014-2016 | 1.25 (0.51-3.03) | 1.37 (0.55-3.41) | 1.30 (0.52-3.25) | NA | 1.21 (0.48-3.05) |
| 2017-2019 | 1.00 | 1.00 | 1.00 | NA | 1.00 |
| Ethnicity: White/Caucasian vs. Other | 0.94 (0.26-3.41) | 1.21 (0.32-4.58) | 1.26 (0.33-4.78) | 1.27 (0.33-4.80) | 1.30 (0.34-4.93) |
| **Environmental & behavioural exposure** |  |  |  |  |  |
| Tobacco use |  |  |  |  |  |
| Never | 1.00 | 1.00 | 1.00 | 1.00 | 1.00 |
| Past | 0.63 (0.17-2.27) | 0.43 (0.11-1.75) | 0.45 (0.11-1.82) | 0.44 (0.11-1.80) | 0.51 (0.12-2.13) |
| Current | 0.79 (0.19-3.32) | 0.73 (0.16-3.21) | 0.74 (0.17-3.28) | 0.72 (0.16-3.27) | 0.85 (0.18-4.01) |
| Marijuana: Current or Past vs. Never | 0.39 (0.15-0.99)* | 0.53 (0.19-1.48) | 0.47 (0.17-1.34) | 0.47 (0.16-1.34) | 0.47 (0.16-1.35) |
| Asbestos: Yes vs. No | 0.44 (0.19-1.02) | 0.43 (0.18-1.06) | 0.43 (0.17-1.05) | 0.42 (0.17-1.04) | 0.42 (0.17-1.04) |
| Alcoholism: Yes vs. No | 1.09 (0.23-5.15) | 1.40 (0.28-6.90) | 1.72 (0.33-9.13) | 1.61 (0.29-8.89) | 1.61 (0.29-8.88) |
| **Comorbidities** |  |  |  |  |  |
| Any comorbidities: At least one vs. None | 2.66 (1.18-5.99)* | 2.08 (0.86-5.02) | 2.08 (0.86-5.03) | 2.07 (0.85-5.02) | 2.03 (0.83-4.96) |
| Cardiovascular diseases: Yes vs. No | 1.99 (0.87-4.52) | 1.55 (0.64-3.72) | 1.56 (0.65-3.76) | 1.62 (0.66-3.93) | 1.91 (0.77-4.73) |
| Respiratory diseases: Yes vs. No | 2.92 (1.20-7.14)* | 2.64 (1.07-6.54)* | 2.62 (1.05-6.52)* | 2.59 (1.04-6.46)* | 2.45 (0.98-6.14) |
| Neoplastic comorbidities: Yes vs. No | 1.56 (0.57-4.29) | 1.22 (0.43-3.48) | 1.22 (0.43-3.50) | 1.22 (0.43-3.49) | 1.35 (0.47-3.91) |
| Renal insufficiency: Yes vs. No | 0.53 (0.14-2.05) | 0.40 (0.10-1.62) | 0.42 (0.10-1.70) | 0.42 (0.10-1.68) | 0.42 (0.10-1.69) |
| Diabetes: Yes vs. No | 1.44 (0.52-4.00) | 1.19 (0.42-3.41) | 1.20 (0.41-3.50) | 1.19 (0.41-3.47) | 1.18 (0.41-3.46) |
| **Clinical status** |  |  |  |  |  |
| Histopathological type: Other vs. NSCLC | 0.62 (0.24-1.56) | 0.65 (0.25-1.68) | NA | 0.66 (0.26-1.69) | 0.69 (0.26-1.79) |
| Stage: I-II vs. III-IV | 2.76 (1.02-7.52)* | 2.37 (0.86-6.58) | 2.36 (0.84-6.69) | 2.44 (0.86-6.98) | 2.00 (0.67-5.97) |
| 1^st^ primary care encounter type |  |  |  |  |  |
| Symptom or sign | 1.00 | 1.00 | 1.00 | 1.00 | 1.00 |
| Radiology request (CT or X-ray) | 0.55 (0.25-1.20) | 0.51 (0.23-1.12) | 0.53 (0.24-1.19) | 0.53 (0.24-1.19) | 0.48 (0.21-1.10) |
| Observed weight loss | 4.28 (0.54-34.18) | 4.07 (0.50-32.91) | 4.10 (0.50-33.36) | 4.07 (0.50-33.10) | 3.82 (0.46-31.44) |
| Thrombocytosis | NA | NA | NA | NA | NA |
| 1^st^ cancer treatment |  |  |  |  |  |
| Systemic therapy | 1.00 | 1.00 | 1.00 | 1.00 | 1.00 |
| Surgery | 2.25 (0.70-7.25) | 1.88 (0.57-6.20) | 1.78 (0.51-6.18) | 1.78 (0.51-6.18) | 1.25 (0.33-4.83) |
| Radiotherapy | 0.98 (0.40-2.43) | 0.73 (0.28-1.89) | 0.66 (0.24-1.78) | 0.66 (0.24-1.78) | 0.66 (0.24-1.78) |
| Systemic therapy+radiotherapy^a^ | 3.60 (0.73-17.85) | 3.26 (0.65-16.41) | 2.98 (0.56-15.87) | 2.93 (0.54-15.78) | 2.92 (0.54-15.81) |

Data in parentheses are 95% confidence intervals.

NSCLC, non-small-cell lung cancer; CT, computed tomography; NA, not applicable.

^*^Statistically significant, as indicated by the p value lower than 0.05.

Adjustment: (model 1) no adjustment; (model 2) sex, age; (model 3) sex, age, histopathology; (model 4) sex, age, histopathology, year of diagnosis; (model 5) sex, age, histopathology, year of diagnosis, ethnicity, hospital.

^a^Both systemic therapy and radiotherapy were initiated on the same day.

Table S13. Subgroup analysis on associations between first primary care encounter types and diagnostic delay (diagnostic interval >35 days) in patients with any thoracic cancer

| **1st primary care encounter** | **Odds ratio**  **(Model 1)** | **Odds ratio**  **(Model 2)** | **Odds ratio**  **(Model 3)** | **Odds ratio**  **(Model 4)** | **Odds ratio**  **(Model 5)** |
| --- | --- | --- | --- | --- | --- |
| Symptom or sign |  |  |  |  |  |
| Pneumonia or infection | 8.44 (1.57-45.39)* | 7.73 (1.41-42.35)* | 7.47 (1.36-41.06)* | 6.47 (1.14-36.61)* | 6.05 (1.04-35.25)* |
| COPD/COAD | NA | NA | NA | NA | NA |
| Pain | 2.81 (0.48-16.43) | 2.56 (0.43-15.29) | 2.17 (0.35-13.66) | 1.89 (0.29-12.33) | 1.71 (0.25-11.52) |
| Cough | 1 | 1 | 1 | 1 | 1 |
| Bronchitis | 3.75 (0.47-29.75) | 3.12 (0.36-26.61) | 2.97 (0.34-25.81) | 2.08 (0.22-19.89) | 1.72 (0.17-17.72) |
| Asthma | 3.13 (0.38-25.57) | 2.94 (0.35-24.43) | 2.80 (0.33-23.59) | 2.48 (0.28-21.52) | 2.39 (0.26-21.70) |
| Other | 2.38 (0.52-10.88) | 2.23 (0.48-10.35) | 2.17 (0.46-10.12) | 1.76 (0.36-8.68) | 1.26 (0.23-6.74) |
|  |  |  |  |  |  |
| Radiology request: CT vs. X-ray | 0.48 (0.20-1.17) | 0.45 (0.18-1.12) | 0.48 (0.19-1.22) | 0.46 (0.18-1.18) | 0.51 (0.19-1.37) |

Data in parentheses are 95% confidence intervals.

COPD, chronic obstructive pulmonary disease; COAD, chronic obstructive airways disease; CT, computed tomography.

^*^Statistically significant, as indicated by the p value lower than 0.05.

Adjustment: (model 1) no adjustment; (model 2) sex, age; (model 3) sex, age, histopathology; (model 4) sex, age, histopathology, year of diagnosis; (model 5) sex, age, histopathology, year of diagnosis, ethnicity, hospital.

Table S14. Subgroup analysis on associations between first primary care encounter types and diagnostic and treatment delay (diagnostic and treatment interval >49 days) in patients with any thoracic cancer

| **1st primary care encounter** | **Odds ratio**  **(Model 1)** | **Odds ratio**  **(Model 2)** | **Odds ratio**  **(Model 3)** | **Odds ratio**  **(Model 4)** | **Odds ratio**  **(Model 5)** |
| --- | --- | --- | --- | --- | --- |
| Symptom or sign |  |  |  |  |  |
| Pneumonia or infection | 11.60 (1.66-81.10)* | 12.01 (1.62-88.74)* | 11.48 (1.54-85.49)* | 9.81 (1.27-76.01)* | 9.52 (1.19-76.13)* |
| COPD/COAD | NA | NA | NA | NA | NA |
| Pain | 4.00 (0.54-29.80) | 4.72 (0.58-38.50) | 3.17 (0.37-27.27) | 2.91 (0.33-25.68) | 2.86 (0.31-26.89) |
| Cough | 1 | 1 | 1 | 1 | 1 |
| Bronchitis | 5.60 (0.47-66.45) | 5.10 (0.38-67.96) | 4.92 (0.36-67.46) | 3.47 (0.22-53.70) | 3.10 (0.19-51.71) |
| Asthma | 4.80 (0.40-58.01) | 5.46 (0.43-69.29) | 5.23 (0.41-67.51) | 4.61 (0.35-61.29) | 4.68 (0.34-64.54) |
| Other | 2.80 (0.57-13.83) | 2.90 (0.56-14.89) | 2.77 (0.54-14.30) | 2.28 (0.41-12.84) | 1.74 (0.28-10.69) |
|  |  |  |  |  |  |
| Radiology request: CT vs. X-ray | 0.71 (0.23-2.17) | 0.68 (0.22-2.11) | 0.64 (0.20-2.01) | 0.64 (0.20-2.05) | 0.69 (0.21-2.24) |

Data in parentheses are 95% confidence intervals.

COPD, chronic obstructive pulmonary disease; COAD, chronic obstructive airways disease; CT, computed tomography.

^*^Statistically significant, as indicated by the p value lower than 0.05.

Adjustment: (model 1) no adjustment; (model 2) sex, age; (model 3) sex, age, histopathology; (model 4) sex, age, histopathology, year of diagnosis; (model 5) sex, age, histopathology, year of diagnosis, ethnicity, hospital.
